# Gastric Cancer Biomarkers: From Cellular Identity to Precision Oncology

**DOI:** 10.3390/cancers18172757

**Published:** 2026-08-25

**Authors:** Catalin-Bogdan Satala, Alina-Mihaela Gurau, Gabriela Patrichi, Gabriela Gurau, Roxana-Cristina Mehedinti, Daniela Mihalache

**Affiliations:** 1 Medical and Pharmaceutical Research Center, Faculty of Medicine and Pharmacy, “Dunarea de Jos” University of Galati, 800008 Galati, Romania; catalin.satala@ugal.ro (C.-B.S.); gabriela.gurau@ugal.ro (G.G.); roxana.mehedinti@ugal.ro (R.-C.M.); daniela.mihalache@ugal.ro (D.M.); 2 Department of Pathology, Clinical County Emergency Hospital Braila, 810325 Braila, Romania; 3 The School for Doctoral Studies in Biomedical Sciences, “Dunarea de Jos” University of Galati, 800008 Galati, Romania; 4 The Doctoral School of Medicine and Pharmacy, “George Emil Palade” University of Medicine, Pharmacy, Science and Technology of Targu Mures, 540142 Targu Mures, Romania; gabriela.constantin@umfst.ro; 5 “Sf. Ioan” Clinical Emergency Pediatric Hospital, 800487 Galati, Romania

**Keywords:** gastric cancer, biomarkers, precision oncology, molecular pathology, tumor evolution, gastric lineage identity, stemness, epithelial–mesenchymal transition, immune evasion, targeted therapy

## Abstract

Gastric Cancer (GC) is characterized by remarkable biological heterogeneity, reflected by the continuous expansion of biomarkers with diagnostic, prognostic, predictive, and therapeutic relevance. However, most reviews organize these biomarkers according to signaling pathways or individual molecular alterations, often overlooking the interconnected biological processes and functional states that shape gastric carcinogenesis and tumor evolution. This review proposes a biology-oriented conceptual framework that interprets GC biomarkers through five fundamental biological questions capturing interconnected biological capabilities and tumor states: preservation of gastric lineage identity, acquisition of stem cell-like properties, epithelial–mesenchymal transition, immune evasion, and dependence on actionable oncogenic pathways. Within each biological domain, biomarkers were critically selected and comparatively evaluated according to their biological significance, pathological applicability, translational relevance, and clinical utility. Core biomarkers central to each biological process are discussed alongside complementary biomarkers that further refine biological interpretation and clinical stratification. Rather than presenting another comprehensive catalogue of molecular alterations, this review integrates current evidence into a coherent framework linking tumor biology with diagnostic pathology and precision oncology. By emphasizing biological capabilities rather than isolated molecular pathways, this approach provides a practical perspective for biomarker interpretation and establishes a conceptual foundation for future multimarker strategies in GC.

## Simple Summary

Gastric cancer (GC) contains remarkable biological diversity, making it difficult to predict tumor behavior and select the most appropriate treatment. Although numerous biomarkers have been identified, they are usually discussed individually or grouped according to molecular pathways, which does not fully reflect how GC develops over time. This review proposes a different perspective by organizing biomarkers according to five fundamental biological questions that capture interconnected dimensions of gastric cancer biology, including gastric lineage identity, stemness, epithelial plasticity, immune evasion, and actionable oncogenic dependencies. Rather than representing a fixed sequence of events, these biological domains interact dynamically throughout tumor development and progression. By integrating biomarkers within a biology-oriented framework, this review provides a more coherent interpretation of tumor progression while linking tumor biology with diagnostic pathology and precision oncology. This approach may facilitate biomarker interpretation, support future multimarker strategies, and offer a practical framework for both researchers and clinicians investigating GC.

## 1. Introduction

Gastric carcinogenesis is not a single molecular event but a dynamic biological process during which gastric epithelial cells progressively lose their original lineage identity while acquiring new functional capabilities that sustain tumor initiation, progression, dissemination, immune escape, and therapeutic resistance [1,2]. Throughout this evolutionary trajectory, distinct molecular alterations may emerge, interact, and evolve over time, generating biomarkers that reflect not only the presence of malignancy but also different biological states and functional properties of the tumor. Consequently, biomarkers have evolved from simple diagnostic adjuncts into fundamental tools for understanding tumor biology, refining pathological classification, improving prognostic stratification, and guiding precision oncology. This conceptual evolution has been accompanied by an extraordinary expansion of the gastric cancer (GC) biomarker landscape. Advances in molecular pathology, next-generation sequencing, transcriptomics, spatial biology, and digital pathology have identified an increasing number of biomarkers associated with epithelial differentiation, stemness, epithelial–mesenchymal transition (EMT), tumor–immune interactions, and oncogenic signaling [1,3]. Simultaneously, precision medicine has fundamentally transformed the clinical role of pathology. Biomarkers such as HER2, PD-L1, microsatellite instability, CLDN18.2, and FGFR2b are no longer regarded solely as biological indicators but have become integral components of therapeutic decision-making, while numerous additional biomarkers continue to emerge as potential diagnostic, prognostic, predictive, and therapeutic candidates [3,4].

Despite this remarkable progress, the rapidly expanding biomarker literature has become increasingly fragmented. Most published reviews organize biomarkers according to signaling pathways, molecular classes, or individual molecules, providing comprehensive summaries of their biological functions and clinical relevance [3,4]. Although these approaches have substantially advanced our understanding of GC biology, they often overlook a more fundamental biological principle: gastric carcinogenesis represents a progressive evolutionary process rather than the independent activation of isolated molecular pathways. Consequently, biomarkers participating in the same biological transition are frequently discussed separately, whereas biologically unrelated molecules are grouped together simply because they belong to a common signaling cascade. Such classifications provide valuable mechanistic information but are often less effective in illustrating how tumor cells progressively acquire the biological capabilities required for malignant progression [3,4].

To address this limitation, the present review proposes a biology-oriented conceptual framework that organizes the current biomarker landscape according to five interconnected biological capabilities and tumor states relevant to gastric carcinogenesis, rather than according to individual signaling pathways or isolated molecular markers. Specifically, the available evidence is structured around five fundamental biological questions that capture distinct but interconnected dimensions of tumor biology: preservation of gastric lineage identity, acquisition of stem cell-like properties, epithelial–mesenchymal transition, immune evasion, and dependence on actionable oncogenic pathways. This perspective allows biomarkers sharing a common biological function to be evaluated comparatively within the context of the cellular process they represent, thereby providing a more integrated interpretation of tumor evolution. Within each biological domain, biomarkers are critically assessed according to predefined criteria encompassing biological relevance, consistency of published evidence, pathological applicability, translational significance, and clinical utility. Rather than presenting another comprehensive catalogue of molecular alterations, this review aims to establish a biologically coherent framework that explains how complementary biomarkers collectively describe the progressive transformation of gastric epithelial cells throughout carcinogenesis. By shifting the focus from individual biomarkers to the biological capabilities they represent, this biology-driven approach provides a conceptual bridge between tumor biology, diagnostic pathology, and precision oncology, while offering a rational foundation for future multimarker strategies in GC.

## 2. Materials and Methods

### 2.1. Study Design

This review was conducted according to a predefined methodological protocol established before literature screening and biomarker selection. The framework was developed to ensure a transparent, reproducible, and biologically coherent evaluation of the available evidence while minimizing selection bias through predefined eligibility criteria, literature search procedures, and biomarker selection principles.

**Figure 1:**
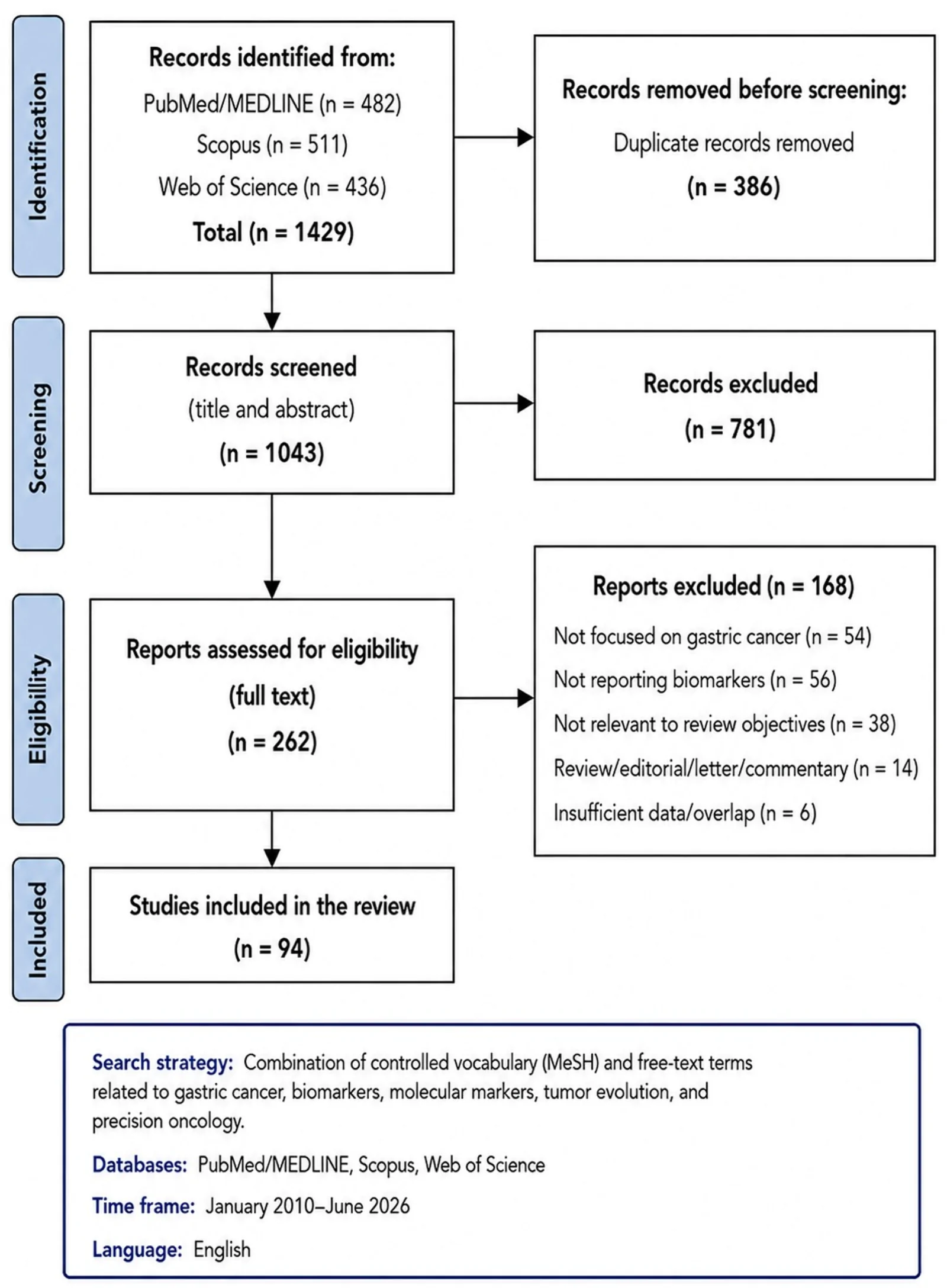
PRISMA 2020 flow diagram of the literature identification and selection process for biomarker studies in gastric cancer.

Rather than providing an exhaustive catalogue of all biomarkers investigated in GC, the primary objective was to identify the biomarkers most consistently supported by the current evidence within five predefined biological domains representing key dimensions of cellular identity during gastric carcinogenesis. Consequently, the review was designed as a structured evidence-based review with comparative evidence synthesis, emphasizing biological relevance, consistency of published data, and clinical applicability. Although the present study was not designed as a formal systematic review or meta-analysis, methodological principles derived from the Preferred Reporting Items for Systematic Reviews and Meta-Analyzes (PRISMA 2020) statement were incorporated whenever applicable to improve transparency in literature identification, study selection, and evidence synthesis (Figure 1).

### 2.2. Development of the Biological Framework

Before initiating the literature search, a biology-oriented conceptual framework was established to guide evidence synthesis throughout the review. Rather than organizing the literature according to isolated signaling pathways or individual biomarkers, the available evidence was structured around five predefined biological questions representing the major biological capabilities progressively acquired during gastric carcinogenesis. The predefined biological questions were: (1) Has the cell preserved its gastric lineage identity? (2) Has the cell acquired stem cell-like properties? (3) Is the cell undergoing epithelial–mesenchymal transition? (4) Can the tumor evade host immune surveillance? and (5) Has the tumor become dependent on actionable oncogenic pathways?

These biological domains were selected because they collectively encompass the principal biological transitions that characterize gastric carcinogenesis, from preservation of lineage identity to the acquisition of therapeutically actionable molecular dependencies. Organizing the literature according to biologically meaningful questions enabled a comparative evaluation of biomarkers fulfilling similar biological functions rather than simply summarizing individual molecular pathways (Table 1).

### 2.3. Literature Search Strategy

A comprehensive literature search was conducted using PubMed/MEDLINE, Scopus, and Web of Science to identify studies addressing biomarkers in gastric cancer, with particular emphasis on biological processes related to gastric lineage identity, stemness and cellular plasticity, epithelial–mesenchymal transition, immune regulation, genomic alterations, and actionable oncogenic pathways. The search strategy combined controlled vocabulary, database-specific indexing terms, and free-text terms related to gastric cancer and biomarker research. For PubMed/MEDLINE, terms related to gastric cancer were combined with terms related to biomarkers and with biomarker-specific terms representing the principal biological domains and biomarkers evaluated in this review.

The corresponding searches in Scopus and Web of Science used the same conceptual domains and biomarker-specific terms, with syntax and searchable fields adapted to the indexing systems of each database. The search covered publications from January 2010 through May 2026. Landmark studies published before 2010 were additionally considered when they represented the original characterization of a biomarker, established a fundamental biological mechanism, or provided essential evidence subsequently validated in human gastric cancer cohorts.

Following the initial literature retrieval, studies were organized according to the five predefined biological domains used as the conceptual framework of the review. Focused searches were subsequently performed within each domain to identify additional evidence addressing individual biomarkers, biological mechanisms, clinicopathological associations, diagnostic or prognostic relevance, predictive value, therapeutic implications, and clinical implementation. Particular attention was given to prospective and randomized clinical studies, regulatory or companion-diagnostic information, and recent clinical developments involving biomarkers with established or emerging therapeutic relevance.

The final database search was performed on 30 May 2026. Database-specific adaptations and the complete PubMed/MEDLINE search strategy are provided in Supplementary Table S1 to facilitate reproducibility.

### 2.4. Study Selection

Eligible publications included clinical trials, original research articles, systematic reviews, and meta-analyzes investigating biomarkers in human gastric adenocarcinoma. Studies were required to provide evidence regarding the biological role of the investigated biomarker together with its diagnostic, prognostic, predictive, or therapeutic significance whenever available. Conference abstracts, editorials, letters to the editor, isolated case reports, duplicate publications, and studies lacking sufficient methodological information were excluded. Experimental studies performed exclusively in cell lines or animal models were considered only when they provided essential mechanistic evidence supporting observations reported in human GC. The study selection process consisted of literature identification, title and abstract screening, full-text eligibility assessment, and final inclusion for qualitative evidence synthesis.

### 2.5. Evidence Assessment and Risk of Bias Considerations

The methodological robustness and clinical relevance of the retrieved evidence were assessed qualitatively during evidence synthesis. Because this study was designed as a structured evidence-based review rather than a formal systematic review or meta-analysis, a single standardized risk-of-bias instrument was not applied across all included publications, given the substantial heterogeneity in study designs, ranging from mechanistic and translational studies to retrospective cohorts, prospective studies, and randomized clinical trials. Instead, the strength of evidence for individual biomarkers was considered according to study design, human validation, consistency of findings across independent cohorts, prospective or randomized clinical validation, and evidence of clinical implementation. Particular attention was given to the potential limitations of retrospective studies, small or single-center cohorts, heterogeneous biomarker assessment methods, variable positivity thresholds, and lack of independent validation. Preclinical findings were considered supportive of biological plausibility but were not regarded as sufficient evidence for clinical utility. Where available, prospective clinical trials, randomized studies, companion diagnostic validation, and regulatory or guideline-based implementation were given greater evidentiary weight. This qualitative assessment was incorporated into the comparative interpretation of biomarkers, while the explicit five-level evidence hierarchy described below was used to characterize their current evidentiary and clinical status (Supplementary Table S2).

For comparative interpretation, the level of evidence supporting each biomarker was classified into five categories according to the highest level of evidence available: (1) preclinical evidence, based predominantly on experimental studies providing mechanistic or biological support; (2) retrospective clinical validation, based on observational studies or retrospective cohorts demonstrating associations with pathological, prognostic, or predictive endpoints; (3) prospective clinical validation, based on prospectively designed clinical studies confirming biomarker performance or clinical relevance; (4) randomized clinical evidence, based on randomized controlled trials demonstrating biomarker-associated treatment benefit or predictive utility; and (5) regulatory or clinical implementation, defined by incorporation of the biomarker into an approved companion diagnostic, regulatory indication, established clinical testing pathway, or recognized clinical guideline. When evidence from multiple categories was available, biomarkers were assigned to the highest applicable category, while the limitations of lower-level or inconsistent evidence were retained in the qualitative interpretation.

### 2.6. Biomarker Selection Strategy

Biomarker selection was performed using a predefined qualitative comparative framework based on five criteria: biological specificity, human evidence, evidence consistency, clinical relevance, and technical feasibility. Biological specificity referred to evidence supporting a direct role within the biological domain under investigation; human evidence referred to validation in gastric adenocarcinoma cohorts; evidence consistency reflected reproducibility across independent studies; clinical relevance encompassed associations with diagnosis, prognosis, prediction, or therapy; and technical feasibility referred to assessment using established pathological or molecular techniques.

These criteria were considered jointly rather than assigned numerical weights or predefined quantitative thresholds. Within each biological domain, two biomarkers were designated as core biomarkers when they provided the most complementary representation of the principal biological features addressed by that domain and were supported by a consistent combination of biological and human evidence. The selection of two core biomarkers per domain was therefore an organizational and comparative decision intended to provide a balanced representation of the five domains, rather than a statistically derived ranking or evidence threshold.

Additional biomarkers with relevant but more limited, overlapping, or emerging evidence were retained as complementary biomarkers. The core/complementary distinction was not intended to imply that only two biomarkers are relevant within each biological domain, but rather to identify representative biomarkers that best illustrate the principal biological dimension while acknowledging the broader biomarker landscape. The resulting selection of core and complementary biomarkers is summarized in Table 2.

The synthesis was comparative rather than purely descriptive: within each biological domain, biomarkers were evaluated in relation to their biological specificity, human validation, consistency of findings, clinical relevance, and technical feasibility, with particular emphasis on complementary versus overlapping information provided by individual biomarkers.

### 2.7. Methodological Considerations

The biomarker prioritization strategy adopted in the present review represents a qualitative evidence synthesis rather than a quantitative ranking of biomarker performance. Accordingly, the designation of core and additional biomarkers should not be interpreted as an absolute hierarchy of diagnostic or prognostic value. Instead, this classification reflects the overall consistency of currently available evidence together with biological relevance and potential clinical applicability within each predefined biological domain. This structured approach was intended to facilitate a biologically coherent interpretation of the expanding GC biomarker literature while providing a transparent rationale for biomarker prioritization and future multimarker panel development.

## 3. Has the Cell Preserved Its Gastric Lineage Identity?

Preservation of gastric lineage identity represents one of the defining biological characteristics of the normal gastric epithelium, reflecting a tightly regulated differentiation program that maintains tissue architecture, secretory function, and epithelial homeostasis. Rather than being determined by a single molecule, gastric identity is sustained through the coordinated expression of lineage-specific structural proteins, secretory products, and differentiation-associated genes that collectively distinguish gastric epithelial cells from intestinal or undifferentiated phenotypes. During gastric carcinogenesis, this lineage program undergoes progressive disruption, accompanied by increasing phenotypic plasticity and dedifferentiation [5,6]. The transition from chronic gastritis to intestinal metaplasia, dysplasia, and ultimately invasive adenocarcinoma is frequently associated with attenuation or loss of gastric-specific differentiation markers, reflecting a gradual erosion of the normal gastric phenotype rather than an abrupt molecular switch. Consequently, preservation of gastric lineage identity has emerged as a biologically meaningful indicator of tumor differentiation and cellular origin, providing insight into the evolutionary trajectory of gastric carcinogenesis beyond conventional histomorphological classification [5,6,7].

Recognition of gastric lineage identity also carries important translational implications. Although histological classifications such as the Lauren system continue to provide valuable clinicopathological information, they do not fully capture the molecular and phenotypic heterogeneity observed within gastric adenocarcinoma [3]. Immunohistochemical biomarkers capable of identifying preservation or loss of gastric differentiation therefore offer a complementary approach for tumor characterization, particularly in morphologically heterogeneous neoplasms. Among the numerous biomarkers investigated over the past two decades, only a limited number have demonstrated consistent biological specificity together with robust clinicopathological validation across independent patient cohorts. Based on the predefined selection criteria adopted in the present review, CLDN18 and VSIG1 emerged as the most representative biomarkers of preserved gastric lineage identity owing to their tissue-restricted expression, reproducible immunohistochemical assessment, and substantial body of translational evidence [8,9]. Additional biomarkers, including MUC5AC, TFF1, MUC6, and pepsinogen C, provide valuable complementary information regarding gastric differentiation, although their expression patterns, clinical validation, or biological specificity are comparatively less consistent. The following sections critically examine the evidence supporting these biomarkers and discuss their respective contributions to defining gastric lineage identity in gastric adenocarcinoma [10].

### 3.1. Claudin-18 (CLDN18)

Among the numerous biomarkers investigated in GC, CLDN18 occupies a unique position because it primarily reflects tissue identity rather than tumor behavior. While most biomarkers are introduced to characterize proliferation, invasion, metastatic potential, or therapeutic response, CLDN18 provides insight into a more fundamental biological question: whether neoplastic cells have retained their original gastric epithelial phenotype. This property makes it particularly relevant to the first biological question addressed in this review—Has the cell preserved its gastric lineage identity? Moreover, CLDN18 represents one of the few biomarkers whose biological significance has evolved seamlessly from lineage identification to direct clinical application. The importance of CLDN18 emerged from studies aimed at identifying tissue-restricted membrane proteins suitable for targeted therapy rather than from conventional biomarker discovery. In their seminal work, Sahin and colleagues identified the CLDN18.2 splice variant as a highly gastric-specific antigen, demonstrating that its expression is almost exclusively confined to differentiated gastric epithelial cells while being virtually absent from other normal tissues [11]. This remarkable tissue specificity immediately distinguished CLDN18.2 from other gastric differentiation markers and suggested that its persistence in neoplastic cells could serve as a reliable indicator of preserved gastric epithelial identity. This concept was subsequently strengthened by large clinicopathological studies demonstrated that CLDN18.2 expression is retained in a substantial proportion of gastric adenocarcinomas despite the profound architectural and molecular changes accompanying malignant transformation [12]. Their findings established an important biological principle: malignant progression does not necessarily imply complete loss of gastric lineage differentiation.

CLDN18 belongs to the claudin family of transmembrane proteins that form the structural backbone of epithelial tight junctions. Two major splice variants have been described, exhibiting distinct tissue distributions. CLDN18.1 is predominantly expressed in pulmonary epithelium, whereas CLDN18.2 is largely restricted to gastric mucosa. Under physiological conditions, CLDN18.2 contributes to epithelial polarity and mucosal barrier integrity, remaining largely concealed within intact tight junctions. As gastric carcinogenesis progresses, disruption of epithelial architecture exposes extracellular domains that are normally inaccessible, creating a unique biological situation in which a lineage-specific molecule simultaneously becomes an attractive therapeutic target. This dual role largely explains why CLDN18 has attracted attention from both pathologists and clinical oncologists [11,13].

The behavior of CLDN18 during gastric carcinogenesis differs substantially from that of biomarkers associated with tumor progression. In normal gastric mucosa, expression is diffuse and strongly membranous, whereas intestinal metaplasia is frequently accompanied by marked reduction or complete loss of staining, reflecting replacement of the gastric differentiation program by an intestinal phenotype. However, many invasive gastric adenocarcinomas continue to express CLDN18, indicating that acquisition of invasive or metastatic potential does not necessarily require complete loss of gastric epithelial identity. This observation is particularly relevant in diffuse-type GCs, where preservation of lineage-specific markers may coexist with highly aggressive biological behavior. Consequently, CLDN18 should not be interpreted as a marker of tumor aggressiveness but rather as an indicator of the extent to which gastric lineage differentiation has been maintained throughout tumor evolution [8,12].

The pathological characteristics of CLDN18 have been refined through a series of large clinicopathological studies. A major step forward was provided by Dottermusch and colleagues, whose whole-slide analysis of 481 gastric adenocarcinomas demonstrated that heterogeneous expression is an intrinsic feature of CLDN18 rather than an occasional observation. Both intertumoral and intratumoral heterogeneity were common findings, highlighting the limitations of small biopsy samples and emphasizing the need for adequate tumor representation during immunohistochemical assessment. These observations also helped explain the variability reported in earlier studies and contributed to the standardization of pathological evaluation. Subsequent investigations expanded the biological context in which CLDN18 should be interpreted [12]. In one of the largest clinicopathological series published to date, Kwak et al. analyzed CLDN18.2 expression in 1000 stage II–IV gastric adenocarcinomas while simultaneously evaluating its relationship with HER2, FGFR2, PD-L1, microsatellite instability, and Epstein–Barr virus status. Rather than identifying a simple association with existing molecular subgroups, the study demonstrated that CLDN18 defines a partially independent biological subset, reinforcing its role as a distinct biomarker within the molecular landscape of GC [14]. Complementary evidence was provided by Pellino and colleagues, who integrated CLDN18 into contemporary molecular pathology algorithms and showed that its evaluation complements established biomarker panels rather than replacing them. Collectively, these studies positioned CLDN18 within the broader framework of precision oncology while preserving its primary significance as a marker of gastric epithelial lineage [15].

The clinical evolution of CLDN18 closely mirrored these biological discoveries. Once disruption of epithelial polarity was recognized to expose the extracellular domain of CLDN18.2, antibody-based targeting became a realistic therapeutic strategy. The FAST trial provided the first prospective evidence that patients with CLDN18.2-positive advanced GC derive clinically meaningful benefit from zolbetuximab combined with chemotherapy. These findings were subsequently confirmed by the pivotal phase III SPOTLIGHT and GLOW trials, which independently demonstrated improvements in progression-free and overall survival among patients with HER2-negative, CLDN18.2-positive gastric or gastro-esophageal junction adenocarcinoma. As a result, CLDN18 has undergone a remarkable transformation from a marker of gastric differentiation to a companion diagnostic that directly influences treatment selection [16,17,18].

Despite these advances, several challenges remain. Reported positivity rates continue to vary across studies owing to differences in antibody clones, scoring systems, positivity thresholds, and specimen selection. Intratumoral heterogeneity further complicates pathological interpretation, particularly in limited biopsy material, where sampling bias may underestimate true expression. These issues have prompted international efforts to harmonize CLDN18 testing, culminating in large implementation studies such as that of Shitara et al., which supported the global prevalence of CLDN18.2 using standardized companion diagnostic methodology and supported the incorporation of CLDN18 testing into routine clinical practice [8,15].

The scientific development of CLDN18 illustrates how the significance of a biomarker can evolve as biological, pathological, and clinical evidence accumulates. Initially recognized as a gastric-specific tight-junction protein, CLDN18 subsequently became a marker of preserved epithelial lineage, was incorporated into contemporary molecular classification strategies, and ultimately emerged as a predictive biomarker guiding targeted therapy. Few biomarkers in GC have completed such a comprehensive translational journey. The current status of CLDN18 as the established biomarker of preserved gastric lineage identity is the result of more than two decades of progressive biological, pathological, and clinical validation [19]. The landmark studies that supported this evidence base are summarized in Table 3.

### 3.2. V-Set and Immunoglobulin Domain Containing 1 (VSIG1)

Among the biomarkers associated with gastric epithelial differentiation, VSIG1 represents one of the most lineage-restricted molecules identified to date. Unlike CLDN18, whose clinical relevance has largely been driven by its therapeutic implications, the significance of VSIG1 lies primarily in its ability to reflect preservation of the gastric epithelial program during tumor evolution. Its expression is closely linked to gastric differentiation, making it particularly valuable for understanding whether malignant cells maintain their original phenotypic identity or progressively undergo lineage reprogramming. For this reason, VSIG1 complements rather than duplicates the information provided by CLDN18, offering an additional perspective on the biological question addressed in this review. The history of VSIG1 began with its identification by Scanlan and colleagues during efforts to characterize tissue-restricted antigens. The protein was recognized as a member of the immunoglobulin superfamily displaying a remarkably selective expression pattern, being largely confined to normal gastric epithelium and testicular tissue [20]. This restricted distribution immediately suggested that VSIG1 might represent a marker of gastric epithelial differentiation rather than a molecule directly involved in malignant transformation. At that stage, however, its biological function remained largely unknown, and its potential role in GC had yet to be explored. Important mechanistic insights emerged several years later from the experimental work of Oidovsambuu and colleagues, who demonstrated that VSIG1 contributes to normal gastric gland development and epithelial organization. Experimental models showed that disruption of VSIG1 expression alters glandular architecture and epithelial differentiation, supporting the concept that the molecule participates in maintaining gastric epithelial homeostasis rather than simply serving as a passive differentiation marker [21,25]. These observations provided the biological rationale for subsequently investigating whether alterations in VSIG1 expression accompany gastric carcinogenesis.

The first clinicopathological evidence linking VSIG1 to GC was provided by Chen et al., who demonstrated that loss of VSIG1 expression is associated with adverse clinicopathological features and reduced patient survival. This study introduced an important concept that continues to influence current interpretation of the marker: decreasing VSIG1 expression reflects progressive loss of gastric epithelial differentiation rather than merely increasing tumor aggressiveness. Although the prognostic implications required further validation, the work supported VSIG1 as a biologically meaningful marker whose expression changes during tumor progression [22]. Subsequent functional studies by Inoue and colleagues reinforced this concept by demonstrating tumor suppressor-like properties of VSIG1 in experimental models while confirming that preserved expression is associated with a more favorable clinical outcome [23]. Together, these investigations shifted the perception of VSIG1 from a tissue-specific protein to a molecule with an active role in maintaining epithelial differentiation.

More recent pathological studies have refined the interpretation of VSIG1 by placing it within the dynamic process of gastric tumor evolution. Rather than behaving as a simple positive-or-negative marker, VSIG1 displays considerable spatial heterogeneity, with progressive loss frequently occurring at the invasive front and in areas showing features of epithelial–mesenchymal transition [9]. These observations indicate that preservation of gastric lineage is not uniform throughout the tumor but may vary according to local biological events occurring during invasion. Consequently, assessment of VSIG1 should consider not only the proportion of positive tumor cells but also the distribution of staining within the neoplasm, as focal loss may provide biologically relevant information regarding tumor progression. An additional advance in understanding the biological significance of VSIG1 came from studies examining its relationship with established markers of gastric and intestinal differentiation. Analysis of the MUC2–MUC5AC–CDX2 axis demonstrated that VSIG1 expression closely parallels the gastric phenotype, whereas its loss frequently accompanies acquisition of intestinal characteristics during gastric carcinogenesis. Importantly, these findings suggested that VSIG1 should not be interpreted simply as a stomach-specific marker but rather as an indicator of preservation of the gastric differentiation program. This distinction is particularly relevant because GCs often display mixed or transitional phenotypes, in which lineage conversion occurs gradually rather than as an abrupt biological event. Within this context, VSIG1 provides information that extends beyond anatomical origin and reflects the differentiation state of tumor cells [24].

Despite these encouraging observations, several limitations should be acknowledged. Compared with CLDN18, the evidence supporting VSIG1 is derived from relatively small clinicopathological cohorts, and independent validation studies remain limited. Standardized scoring criteria have not yet been universally adopted, while intratumoral heterogeneity may influence interpretation, particularly in biopsy specimens. Moreover, although accumulating evidence suggests that VSIG1 has tumor suppressor-like properties, the molecular mechanisms responsible for its loss during gastric carcinogenesis remain incompletely understood. These limitations currently preclude its use as a predictive biomarker but do not diminish its value as a marker of gastric epithelial differentiation.

The evolution of VSIG1 over the past two decades illustrates how a lineage-associated molecule can progressively acquire biological and pathological significance through experimental and clinicopathological investigation. From its initial identification as a gastric-restricted protein to its current recognition as a marker reflecting preservation of the gastric differentiation program, each major study has contributed to refining its biological interpretation. Although the available evidence remains less extensive than that supporting CLDN18, the consistency of these findings across experimental models and human GCs justifies the inclusion of VSIG1 among the core biomarkers of preserved gastric lineage identity [9,20,21,22,23,26]. The landmark studies that established the current understanding of VSIG1 are summarized in Table 3.

### 3.3. Additional Markers of Gastric Epithelial Differentiation

Although CLDN18 and VSIG1 represent the most informative biomarkers for assessing preservation of gastric lineage identity, several additional molecules contribute to the characterization of gastric epithelial differentiation. Most of these proteins are physiological components of the gastric mucosa and display expression patterns that closely mirror the differentiation status of gastric epithelial cells. However, compared with CLDN18 and VSIG1, they generally exhibit lower tissue specificity, greater biological variability, or more limited validation in GC, which explains why they are better regarded as complementary rather than core biomarkers. Among these, MUC5AC is one of the best-established markers of foveolar differentiation. Expressed in the superficial gastric epithelium, it is frequently retained in gastric adenocarcinomas with a gastric mucin phenotype, while progressive loss of expression often accompanies intestinal metaplasia and intestinal-type differentiation. Nevertheless, MUC5AC expression is highly heterogeneous and may also be observed in several non-gastric adenocarcinomas, limiting its specificity as an indicator of preserved gastric lineage [10,24].

MUC6, normally expressed by mucous neck cells and pyloric glands, provides complementary information by reflecting differentiation toward deeper gastric glandular compartments. Similar to MUC5AC, its expression is frequently preserved in gastric-type adenocarcinomas but tends to decrease during intestinal phenotypic transition. Combined evaluation of MUC5AC and MUC6 has therefore been used to define gastric mucin phenotypes, although neither marker alone reliably distinguishes preserved gastric identity from broader patterns of mucin production. Another marker closely associated with gastric differentiation is trefoil factor 1 (TFF1), a secretory peptide produced by surface foveolar cells that contributes to mucosal protection and epithelial repair. Experimental studies suggest that TFF1 also functions as a tumor suppressor, and loss of expression is considered an early event during gastric carcinogenesis. Reduced TFF1 expression has been associated with progression along the Correa cascade, supporting its value as an indicator of disruption of the normal gastric differentiation program. However, because TFF1 is influenced by inflammatory signaling, epigenetic alterations, and tumor progression, its expression reflects multiple biological processes beyond lineage preservation alone [10,27].

Pepsinogen C (PGC) represents another classical marker of mature gastric differentiation. Restricted to differentiated gastric glands under physiological conditions, PGC expression progressively declines during chronic atrophic gastritis, intestinal metaplasia, and gastric carcinogenesis, paralleling the loss of normal glandular architecture. Although its high tissue specificity makes PGC an attractive indicator of gastric epithelial maturation, its diagnostic use has gradually diminished owing to variable expression in advanced tumors and the wider availability of more robust immunohistochemical markers [28].

Collectively, these biomarkers provide valuable information regarding the differentiation status of gastric epithelial cells and remain useful components of immunohistochemical panels investigating gastric phenotype. Their greatest strength lies in their complementary interpretation, particularly when evaluated alongside lineage-defining biomarkers such as CLDN18 and VSIG1. Within this framework, MUC5AC, MUC6, TFF1, and PGC refine the assessment of gastric differentiation, whereas CLDN18 and VSIG1 provide the most robust evidence that gastric lineage identity has been preserved during tumor evolution [10,24,27,28].

## 4. Has the Cell Acquired Stem Cell-like Properties?

Preservation of gastric lineage identity provides only a partial understanding of GC biology. As tumor evolution progresses, neoplastic cells may retain features of their tissue of origin while simultaneously acquiring new biological traits that enhance their adaptability and survival. Among these, stem cell-like properties have emerged as one of the defining hallmarks of GC progression, contributing to tumor initiation, phenotypic plasticity, metastatic dissemination, therapeutic resistance, and disease recurrence. Consequently, once the degree of gastric lineage preservation has been established, the next biological question is whether tumor cells have acquired characteristics typically associated with stem cell populations. Unlike normal gastric stem cells, which maintain epithelial homeostasis under tightly regulated physiological conditions, cancer stem-like cells constitute a dynamic and heterogeneous subpopulation capable of self-renewal, multilineage differentiation, and adaptation to environmental stress. Importantly, stemness is now regarded as a plastic rather than a static cellular state. Experimental evidence indicates that differentiated tumor cells may reacquire stem-like characteristics in response to genetic alterations, signaling pathway activation, interactions with the tumor microenvironment, or therapeutic pressure. This dynamic behavior contributes substantially to the intratumoral heterogeneity that characterizes GC and partly explains why tumors with similar histopathological features may follow markedly different clinical courses [29,30].

Numerous biomarkers have been proposed to identify stem cell-like populations in GC, including LGR5, CD44, CD44 variant isoforms, ALDH1, SOX2, OCT4, NANOG, BMI1, and EpCAM. However, the biological and clinical evidence supporting these markers is highly variable. Some primarily identify normal gastric stem-cell populations, whereas others reflect stemness acquired during tumor progression or treatment resistance. Considering the strength of experimental evidence, pathological validation, and consistency across independent studies, LGR5 and CD44v9 emerge as the two biomarkers that most comprehensively capture these complementary aspects of stemness. The remaining markers provide valuable additional information but generally represent specific functional dimensions of the stem-like phenotype rather than its overall biological framework [31,32,33].

### 4.1. Leucine-Rich Repeat-Containing G-Protein-Coupled Receptor 5 (LGR5)

Among the numerous biomarkers proposed to identify stem cell-like populations in GC, LGR5 (leucine-rich repeat-containing G-protein-coupled receptor 5) occupies a unique position because it was not originally discovered as a cancer biomarker. Instead, LGR5 emerged from studies investigating the physiological organization of adult tissue stem cells, where it was identified as a marker of long-lived, self-renewing stem cells responsible for maintaining epithelial homeostasis. Consequently, the biological significance of LGR5 in GC is rooted in normal stem-cell biology rather than in tumor-associated expression alone, making it one of the most biologically grounded markers of stemness currently available. The pivotal advance came in 2010, when Barker and colleagues identified LGR5-positive cells at the base of pyloric glands and demonstrated, through lineage-tracing experiments, that these cells possess long-term self-renewal capacity and generate all differentiated epithelial lineages of the gastric unit. This landmark study established LGR5 as the first bona fide marker of adult gastric stem cells and fundamentally changed the understanding of gastric epithelial maintenance by demonstrating that continuous tissue renewal originates from a discrete population of multipotent stem cells. Importantly, the development of long-lived gastric organoids derived from single LGR5-positive cells further confirmed their remarkable regenerative potential and provided an experimental platform that would later become instrumental for studying gastric carcinogenesis [32,34].

Following the identification of LGR5-positive gastric stem cells, subsequent investigations focused on understanding how these cells maintain epithelial homeostasis. Experimental studies demonstrated that LGR5-positive stem cells undergo predominantly symmetric divisions and compete through a process of neutral drift, whereby individual stem-cell clones are continuously replaced while preserving the overall architecture of the gastric gland. These observations established that stem-cell maintenance is a highly dynamic process rather than the result of a permanently fixed cellular hierarchy. Such findings also suggested that mutations arising within long-lived LGR5-positive stem cells could progressively dominate the stem-cell compartment, thereby providing a plausible biological mechanism for the accumulation of oncogenic alterations during gastric carcinogenesis. The transition from physiological stem-cell biology to GC research occurred rapidly once LGR5 expression was investigated in human gastric tissues. Early clinicopathological studies demonstrated that LGR5 expression progressively increases along the sequence from intestinal metaplasia to dysplasia and invasive adenocarcinoma, indicating that expansion of LGR5-positive cells accompanies the earliest stages of malignant transformation. Rather than representing a marker expressed exclusively by advanced cancers, LGR5 appeared to identify cellular populations already enriched during precursor lesions, supporting the concept that deregulation of normal stem-cell compartments is an early event in gastric tumorigenesis. Additional studies further demonstrated that LGR5 expression is maintained in a subset of gastric adenocarcinomas, frequently correlating with other stemness-associated markers and clinicopathological features indicative of tumor progression. Although reported associations with prognosis have varied across different cohorts, these investigations collectively established LGR5 as one of the principal markers linking normal gastric stem-cell biology with human GC [34,35].

Perhaps the strongest evidence supporting the biological importance of LGR5 emerged from genetically engineered mouse models. Using lineage-tracing strategies combined with conditional inactivation of tumor suppressor pathways, Li and colleagues demonstrated that LGR5-positive gastric stem cells can function as the cell of origin for invasive intestinal-type gastric adenocarcinoma. This finding represented a major conceptual advance because it moved beyond simple expression analysis to establish a direct causal relationship between normal gastric stem cells and malignant transformation. Rather than merely identifying cells exhibiting stem-like characteristics, LGR5-positive cells were shown to possess the capacity to initiate GC following acquisition of appropriate oncogenic alterations. This distinction is particularly important, as relatively few proposed cancer stem-cell markers have been supported by comparable experimental evidence demonstrating their direct involvement in tumor initiation [36,37].

The biological relevance of LGR5 extends beyond tumor initiation. Because LGR5 functions as a receptor within the Wnt signaling pathway, its expression reflects activation of one of the central regulatory networks governing stem-cell maintenance, proliferation, and differentiation. Aberrant activation of Wnt signaling has long been recognized as an important mechanism contributing to gastric carcinogenesis, and persistence of LGR5-positive tumor cell populations is therefore thought to reflect maintenance of a stem-like cellular program rather than simple phenotypic heterogeneity. This concept is consistent with increasing recognition that stemness represents a dynamic and reversible cellular state, allowing differentiated tumor cells to reacquire stem-cell properties in response to genetic evolution or environmental cues. Consequently, LGR5 should be regarded less as a static immunohistochemical marker and more as an indicator of activation of a broader biological program associated with self-renewal and cellular plasticity [36,38,39].

Nevertheless, several limitations should be acknowledged. Unlike markers that identify differentiated gastric epithelial lineages, LGR5 expression is often heterogeneous within individual tumors, and differences in detection methods, antibody performance, RNA-based assays, and scoring systems have contributed to variability among published studies. Furthermore, although numerous investigations have associated LGR5 expression with tumor aggressiveness, lymph node metastasis, or reduced survival, these correlations have not been entirely consistent across independent cohorts, suggesting that LGR5 alone cannot fully capture the complexity of stemness in GC. Instead, its greatest strength lies in providing a biologically validated link between normal gastric stem cells and tumor evolution, rather than serving as a standalone prognostic biomarker [40].

Viewed together, the evidence accumulated over the past decade positions LGR5 as the most biologically validated marker of gastric stem-cell identity and one of the strongest indicators of stem cell-like properties in GC. From its discovery as a marker of normal gastric stem cells to experimental demonstration of its role as a potential cell of origin for gastric adenocarcinoma, LGR5 has progressively evolved from a physiological stem-cell marker into a central component of contemporary models of gastric carcinogenesis. The landmark studies that established this conceptual framework are summarized in Table 4.

### 4.2. CD44v9

Unlike LGR5, which reflects the biological identity of the normal gastric stem-cell compartment, CD44v9 is primarily associated with the persistence of stem-like tumor cells during cancer progression. Its significance lies not in identifying the cells from which GC originates, but in recognizing those that successfully adapt to oxidative stress, survive therapeutic pressure, and sustain tumor growth over time. This functional distinction has made CD44v9 one of the most informative biomarkers for evaluating the maintenance of stem cell-like properties in GC. The origins of this concept can be traced to the landmark study by Takaishi and colleagues, who first identified a population of CD44-positive GC cells capable of self-renewal, sphere formation, and serial tumor initiation following xenotransplantation. Their work provided the first convincing demonstration that GCs contain a subpopulation of tumor-initiating cells with stem-like behavior. Although CD44 variant isoforms were not investigated separately, this study established CD44 as the biological foundation of the GC stem-cell concept and opened the way for subsequent research focusing on individual splice variants [41,42].

A major advance came with the realization that the biological activity of CD44 depends on its alternative splice variants. Among these, CD44v9 rapidly emerged as the most relevant isoform in GC after mechanistic studies demonstrated its interaction with the cystine–glutamate transporter xCT. By stabilizing xCT at the cell membrane, CD44v9 promotes cystine uptake and glutathione synthesis, thereby protecting tumor cells from excessive reactive oxygen species accumulation. This finding fundamentally changed the interpretation of CD44v9. Instead of serving solely as a phenotypic marker of cancer stem-like cells, CD44v9 was shown to participate directly in one of the key mechanisms responsible for their long-term survival. Stemness was no longer viewed simply as a matter of cellular identity but also as the ability to withstand metabolic and oxidative stress. This mechanistic model was soon supported by observations in human GC. Clinical studies evaluating early gastric carcinoma demonstrated that CD44v9 expression identifies patients at increased risk of metachronous recurrence following curative endoscopic resection. The association was particularly intriguing because recurrence occurred despite apparently complete tumor removal, suggesting that CD44v9-positive cells possess biological properties that enable them to survive treatment and contribute to disease re-establishment. These findings represented one of the first practical demonstrations that a stemness-associated biomarker could provide clinically meaningful information beyond conventional histopathological assessment. Subsequent clinicopathological investigations extended these observations in larger patient cohorts. CD44v9 expression was found to increase during gastric carcinogenesis and to persist in a substantial subset of established gastric adenocarcinomas. Several studies also reported associations with deeper tumor invasion, lymph node metastasis, advanced pathological stage, and poorer clinical outcome, although the strength of these correlations varied between cohorts. Despite this variability, the overall body of evidence consistently indicates that CD44v9 expression accompanies biologically aggressive tumor behavior rather than representing an incidental molecular alteration [41,42,43,44].

Further insight came from studies examining the invasive tumor front, where stem-like tumor cells encounter continuous environmental stress and interact dynamically with the surrounding stroma. CD44v9-positive cells were found to accumulate preferentially within these regions, supporting the idea that stemness is closely linked to cellular plasticity and adaptation during tumor invasion. Their localization at the advancing edge of the tumor reinforces the view that CD44v9 identifies a biologically active subpopulation involved in disease progression rather than simply marking proliferating tumor cells. More recent translational studies have strengthened this biological framework by demonstrating that CD44v9 contributes directly to treatment resistance. Experimental inhibition of the CD44v9–xCT axis increases intracellular oxidative stress, reduces the survival of stem-like tumor cells, and enhances sensitivity to fluoropyrimidine-based chemotherapy. Parallel analyzes in patient cohorts have shown that persistent CD44v9 expression is associated with inferior therapeutic response and less favorable survival, indicating that the biological program identified by CD44v9 remains active throughout the course of disease. These observations have also stimulated interest in targeting redox regulation as a therapeutic strategy aimed at eliminating therapy-resistant stem-like tumor cells [45,46].

Several limitations should nevertheless be considered when interpreting CD44v9. Published studies have used different antibodies, scoring systems, and positivity thresholds, making direct comparison between cohorts difficult. In addition, CD44v9 reflects only one component of the stem-like phenotype. While it provides valuable information on oxidative stress resistance and long-term cellular persistence, it does not directly identify the developmental origin of stem-like cells or the signaling pathways responsible for their initial emergence. Its biological value therefore lies in defining how stem-like tumor cells survive rather than how they first arise. The scientific development of CD44v9 illustrates how a surface marker can evolve into a functional biomarker with clear biological significance. The progression from the original identification of CD44-positive tumor-initiating cells to the discovery of the CD44v9–xCT axis and its subsequent clinical validation has established CD44v9 as one of the strongest indicators of stem cell-like persistence in GC. Viewed alongside LGR5, the two biomarkers describe complementary aspects of stemness: LGR5 reflects the stem-cell compartment from which tumor evolution may originate, whereas CD44v9 identifies the mechanisms that allow stem-like tumor cells to endure during progression and treatment [41,42,43,44,45,46]. The landmark studies supporting this biological framework are summarized in Table 4.

### 4.3. Additional Stemness-Associated Biomarkers

Although LGR5 and CD44v9 provide the most comprehensive assessment of stem cell-like properties in GC, several additional biomarkers contribute valuable information regarding specific aspects of the stemness program. Most of these markers are involved in the regulation of self-renewal, pluripotency, cellular plasticity, or resistance to therapy, although the supporting evidence is generally less extensive or less biologically integrated than that available for LGR5 and CD44v9. ALDH1 (aldehyde dehydrogenase 1) is one of the most widely investigated cancer stem cell markers across multiple tumor types. In GC, increased ALDH1 expression has been associated with tumor progression, lymph node metastasis, chemoresistance, and reduced survival. Beyond its role as a marker, ALDH1 participates in cellular detoxification and protection against oxidative stress, thereby supporting the survival of stem-like tumor cells. Nevertheless, unlike LGR5, ALDH1 does not identify a physiological gastric stem-cell population, and its expression is not restricted to cancer stem cells, limiting its specificity. Among transcription factors associated with pluripotency, SOX2 has received considerable attention because of its dual role in gastric epithelial differentiation and stemness regulation. Experimental studies have shown that SOX2 contributes to self-renewal, epithelial plasticity, and tumor progression, while clinicopathological investigations have linked its expression to aggressive disease in selected patient cohorts. However, reported findings remain inconsistent, suggesting that SOX2 is more likely to reflect context-dependent transcriptional reprogramming than a universal stemness marker [47,48,49].

The pluripotency-associated transcription factors OCT4 and NANOG have also been detected in GC and are frequently co-expressed within stem-like tumor cell populations. Their expression has been associated with enhanced tumor initiation, epithelial–mesenchymal plasticity, metastatic potential, and resistance to chemotherapy. Despite these observations, most available evidence derives from experimental models or relatively small clinical series, and neither marker has achieved the level of pathological validation observed for LGR5 or CD44v9. Another marker attracting increasing interest is BMI1, a member of the Polycomb Repressive Complex 1 that plays an essential role in stem-cell self-renewal through epigenetic regulation. In GC, BMI1 overexpression has been associated with tumor progression, maintenance of stem-like phenotypes, and poor clinical outcome. Experimental studies further suggest that BMI1 contributes to treatment resistance by preserving the long-term proliferative capacity of cancer stem-like cells. However, its expression reflects broader epigenetic programs involved in tumor evolution rather than stemness alone [31,48,49].

These biomarkers highlight the biological complexity of stemness in GC. Rather than representing interchangeable indicators of a single cellular state, they capture different dimensions of the stemness program, including metabolic adaptation (ALDH1), transcriptional regulation of pluripotency (SOX2, OCT4, and NANOG), and epigenetic maintenance of self-renewal (BMI1). Within this broader network, LGR5 and CD44v9 remain the most biologically complementary core biomarkers, integrating the developmental origin of stem-like cells with the mechanisms that sustain their persistence throughout tumor progression [30,34,42,46].

## 5. Is the Cell Undergoing Epithelial–Mesenchymal Transition?

The acquisition of stem cell-like properties provides tumor cells with the capacity for self-renewal, phenotypic plasticity, and long-term persistence, yet these characteristics alone do not account for the invasive behavior that defines advanced GC. For tumor progression to occur, neoplastic cells must overcome the structural constraints imposed by epithelial organization, detach from neighbouring cells, remodel their interactions with the extracellular matrix, and acquire the ability to migrate into surrounding tissues. This transition from a relatively confined epithelial phenotype to a more motile and invasive cellular state is orchestrated through epithelial–mesenchymal transition (EMT), a dynamic biological program that has become a central concept in GC progression.

Rather than representing a binary switch, EMT is now understood as a continuum of intermediate cellular states in which epithelial and mesenchymal characteristics coexist. This plasticity enables tumor cells to adapt to changing microenvironmental conditions, facilitates local invasion and metastatic dissemination, and contributes to immune evasion and therapeutic resistance. Although EMT involves a complex network of signaling pathways and transcriptional regulators, a limited number of biomarkers have consistently emerged as reliable indicators of this process. Among them, E-cadherin and SNAIL occupy complementary biological positions. While E-cadherin reflects the integrity of the epithelial phenotype and cell–cell adhesion, SNAIL functions as one of the principal transcriptional repressors driving epithelial dedifferentiation and EMT initiation. Together, these biomarkers capture both the structural consequences and the molecular mechanisms of epithelial–mesenchymal transition, providing a biologically coherent framework for evaluating tumor plasticity in GC [50,51].

### 5.1. E-Cadherin

Among the numerous molecular alterations associated with epithelial–mesenchymal transition (EMT), E-cadherin occupies a unique position because it represents the defining structural component of the epithelial phenotype. Encoded by the CDH1 gene, E-cadherin is the principal adhesion molecule of adherens junctions, maintaining epithelial architecture, apico-basal polarity, and tissue cohesion. The loss of E-cadherin function therefore extends far beyond the disappearance of a membrane protein; it signifies the breakdown of the epithelial program that normally constrains cellular motility and preserves tissue organization. For this reason, reduced membranous E-cadherin expression has become one of the most widely accepted indicators of EMT in GC. The biological importance of E-cadherin in gastric carcinogenesis first emerged through genetic studies demonstrating somatic CDH1 alterations in diffuse-type gastric carcinoma. Becker and colleagues provided the first evidence that mutations affecting the CDH1 gene disrupt epithelial cell cohesion and contribute directly to the characteristic discohesive growth pattern of diffuse GC. Although these observations preceded the widespread recognition of EMT as a biological process, they established the fundamental concept that impairment of cell–cell adhesion represents a driving force in gastric tumor progression rather than a secondary consequence of malignant transformation [50,52].

Subsequent clinicopathological investigations confirmed that the consequences of E-cadherin dysfunction could be appreciated at the protein level. Machado and colleagues demonstrated that reduced or aberrant membranous expression correlated with diffuse histology, lymph node metastasis, advanced tumor stage, and other indicators of aggressive biological behavior. These findings established immunohistochemical evaluation of E-cadherin as a practical surrogate of impaired epithelial cohesion and highlighted its close relationship with tumor invasion and progression. As molecular studies expanded, it became evident that structural mutations explain only a proportion of E-cadherin deficiency in sporadic GC. Epigenetic silencing through CDH1 promoter hypermethylation was subsequently identified as an alternative mechanism responsible for loss of gene expression, demonstrating that multiple molecular events converge on the same biological outcome: disruption of epithelial integrity and increased cellular plasticity. This convergence explains why reduced E-cadherin expression is observed across a broad spectrum of GCs despite considerable molecular heterogeneity [53,54].

Beyond its role in sporadic gastric carcinogenesis, E-cadherin is classically associated with hereditary diffuse GC (HDGC), where germline CDH1 mutations constitute the defining molecular abnormality. Although hereditary disease accounts for only a small proportion of GCs, studies of HDGC have provided fundamental insights into the biological consequences of E-cadherin deficiency, firmly establishing CDH1 as a tumor suppressor gene in GC. Nevertheless, the clinical significance of E-cadherin extends well beyond hereditary disease. In sporadic GC, somatic mutations, promoter hypermethylation, and transcriptional repression all contribute to reduced E-cadherin expression, reinforcing its central role in tumor progression and EMT.

The clinical relevance of these molecular alterations was further strengthened by integrated analyzes combining genetic, epigenetic, and immunohistochemical data. Corso and colleagues demonstrated that disruption of the CDH1 pathway is associated with poorer patient survival, indicating that E-cadherin loss reflects biologically aggressive disease rather than simply defining a particular histological subtype. Importantly, the prognostic value of E-cadherin emerged from the cumulative effects of multiple mechanisms of inactivation, emphasizing that functional disruption of epithelial adhesion, rather than any individual molecular event, underlies its biological significance [55].

The genomic era further expanded this concept. The comprehensive molecular characterization performed by The Cancer Genome Atlas (TCGA) identified CDH1 alterations as one of the defining features of the genomically stable (GS) subtype of gastric adenocarcinoma, a molecular class enriched for diffuse-type tumors. This classification integrated decades of genetic and pathological observations into a unified molecular framework, demonstrating that defective epithelial adhesion is not merely a microscopic feature but a defining characteristic of a distinct biological subtype of GC. More recent mechanistic studies have refined the understanding of how E-cadherin dysfunction promotes tumor invasion. Rather than acting exclusively through passive loss of intercellular adhesion, impaired E-cadherin signaling actively remodels interactions between tumor cells and the extracellular matrix. Experimental evidence has shown that E-cadherin dysfunction enhances integrin β1-dependent cell–matrix adhesion, facilitating cellular migration and invasive behavior. These findings reinforce the concept that E-cadherin loss actively contributes to EMT-associated plasticity rather than simply accompanying tumor progression [56,57].

Despite its established value, E-cadherin should not be interpreted as a standalone marker of EMT. Loss of membranous expression may result from diverse genetic, epigenetic, and transcriptional mechanisms, while partial reduction of expression frequently characterizes intermediate epithelial–mesenchymal states rather than complete mesenchymal conversion. Moreover, intratumoral heterogeneity often leads to regional differences in staining intensity, particularly at the invasive front where EMT-related changes are most pronounced. Consequently, the biological interpretation of E-cadherin is strengthened when it is evaluated together with biomarkers that directly regulate its expression.

Over the past three decades, E-cadherin has evolved from a structural adhesion molecule into one of the best-characterized biomarkers of epithelial integrity in GC. Genetic, epigenetic, clinicopathological, molecular, and functional studies have consistently demonstrated that disruption of the CDH1–E-cadherin axis represents a pivotal step in the acquisition of tumor invasiveness. While E-cadherin captures the structural manifestation of EMT, the molecular events responsible for suppressing its expression are orchestrated by a network of transcriptional regulators, among which SNAIL has emerged as one of the principal drivers of epithelial dedifferentiation [51,54,55,57]. The landmark studies supporting the selection of E-cadherin as a core biomarker of epithelial–mesenchymal transition are summarized in Table 5.

### 5.2. SNAIL

Whereas E-cadherin represents the structural hallmark of epithelial integrity, its loss is rarely a spontaneous event. Instead, it results from coordinated transcriptional programs that actively suppress epithelial differentiation while promoting cellular plasticity. Among the transcription factors orchestrating this transition, SNAIL (SNAI1) occupies a central position. Initially identified as a developmental regulator controlling gastrulation and neural crest migration, SNAIL subsequently emerged as one of the principal molecular drivers of epithelial–mesenchymal transition (EMT) in epithelial malignancies. In GC, its importance extends beyond the transcriptional repression of CDH1, encompassing the coordinated regulation of invasion, metastatic dissemination, stem cell-like properties and therapeutic resistance [58,59,60].

The role of SNAIL in GC was first established by Rosivatz et al. in 2002, who performed the first dedicated investigation of the major EMT transcription factors in primary gastric carcinomas [58]. By simultaneously analyzing SNAIL, SIP1 and TWIST together with E-cadherin, the authors demonstrated that increased SNAIL expression was significantly associated with reduced E-cadherin levels, particularly in diffuse-type gastric carcinoma. More importantly, this study fundamentally changed the understanding of EMT in GC. Rather than viewing E-cadherin loss as an isolated molecular abnormality, the authors demonstrated that it represented the consequence of an active transcriptional program driven by specific regulatory factors. SNAIL therefore emerged as one of the earliest identified upstream regulators linking epithelial dedifferentiation with tumor invasion, establishing a mechanistic framework that has remained central to EMT research ever since. Subsequent work further refined this concept by demonstrating that the biological activity of SNAIL depends not only on its expression but also on its intracellular localisation. As a zinc-finger transcription factor, SNAIL exerts its function only after translocation into the nucleus, where it represses the transcription of epithelial genes, most notably CDH1. Accordingly, Rosivatz and colleagues later showed that nuclear SNAIL expression, rather than overall protein abundance alone, was preferentially associated with E-cadherin loss and aggressive tumor behavior in upper gastrointestinal adenocarcinomas, highlighting that the functional activation of SNAIL is determined by its subcellular localisation. These observations reinforced the concept that EMT is governed by dynamic transcriptional regulation rather than by static alterations in epithelial marker expression [58].

The biological significance of SNAIL was substantially expanded a decade later. In 2012, He et al. demonstrated that increased SNAIL expression independently predicted poor overall survival in patients with GC while simultaneously enhancing tumor cell migration and invasion in experimental models [60]. Importantly, their study also linked SNAIL with the acquisition of stem cell-like characteristics, suggesting that EMT and cancer stemness represent interconnected biological programs rather than independent processes. This finding provides a natural conceptual bridge with the previous section of this review, where LGR5 and CD44v9 were discussed as markers of GC stemness. Rather than acting solely as a regulator of cell motility, SNAIL appears to facilitate the emergence of a highly plastic cellular state capable of simultaneously sustaining invasion, self-renewal and tumor progression. Evidence supporting the clinical relevance of SNAIL was further strengthened by the comprehensive study of Shin et al., also published in 2012. Analyzing tissue microarrays from 314 gastric adenocarcinomas, the investigators demonstrated that nuclear SNAIL overexpression correlated with lymph node metastasis, lymphovascular invasion, perineural invasion, advanced pathological stage and reduced overall survival. Complementary functional experiments showed that SNAIL directly enhanced the migratory and invasive capacities of GC cells while promoting the expression of invasion-related molecules such as VEGF and MMP11. Together, these findings provided one of the strongest clinicopathological validations of SNAIL as a biomarker of aggressive tumor behavior and firmly established its prognostic significance in GC [60,61].

Although the number of studies investigating SNAIL as an isolated biomarker has decreased during the past decade, this trend does not reflect a diminished biological importance. Rather, as the understanding of EMT has evolved, research has progressively shifted towards the complex regulatory networks that converge on SNAIL or are orchestrated by it, positioning this transcription factor as a central molecular hub rather than an individual biomarker. Consequently, contemporary studies more frequently investigate signaling pathways such as TGF-β, WNT/β-catenin, PI3K/AKT, NF-κB, hypoxia-related signaling, or non-coding RNAs that ultimately promote epithelial plasticity through modulation of SNAIL activity. In this context, SNAIL has become less the primary object of investigation and more the common downstream effector through which multiple oncogenic pathways induce EMT. Despite this shift in research focus, contemporary clinical evidence confirms that the biological and prognostic relevance of SNAIL remains intact. In a recent study of patients with advanced GC, Wu et al. (2024) demonstrated that SNAIL overexpression in both primary tumors and gastric mesenteric tumor deposits was associated with lymph node metastasis, tumor deposits and shorter disease-free survival, supporting its continued involvement in EMT-driven tumor dissemination even at advanced stages of disease [62]. Rather than indicating a decline in importance, the progressive integration of SNAIL into broader EMT regulatory networks reflects the maturation of the field, where SNAIL is now regarded as one of the master regulators coordinating epithelial plasticity and metastatic competence in GC [62].

The transcriptional program initiated by SNAIL, however, represents only one component of the EMT regulatory network. Additional transcription factors and structural proteins—including ZEB1, TWIST1, N-cadherin, vimentin and β-catenin—cooperate with SNAIL to stabilise mesenchymal differentiation, reinforce cellular plasticity and facilitate metastatic dissemination. Although these molecules have generally been investigated less extensively as standalone biomarkers in GC, they provide important complementary insights into the complexity and dynamic regulation of EMT and therefore warrant brief consideration [62]. The landmark studies supporting the selection of SNAIL as a core biomarker of epithelial–mesenchymal transition are summarized in Table 5.

### 5.3. Additional Biomarkers of Epithelial–Mesenchymal Transition

Although E-cadherin and SNAIL represent the principal biomarkers of EMT in GC, epithelial plasticity is coordinated by a broader network of transcription factors and structural proteins. Among these, ZEB1, TWIST1, N-cadherin, vimentin, and β-catenin have been the most extensively investigated, complementing the information provided by the two core biomarkers. Like SNAIL, ZEB1 functions as a transcriptional repressor of CDH1, reinforcing the loss of epithelial differentiation and promoting a stable mesenchymal phenotype. In GC, increased ZEB1 expression has been associated with diffuse histology, lymph node metastasis, advanced tumor stage, and poor prognosis. Moreover, its reciprocal interaction with the miR-200 family represents one of the best-characterized regulatory circuits controlling EMT plasticity [50,63].

Another important EMT regulator is TWIST1, which cooperates with SNAIL to suppress epithelial differentiation while promoting invasion, migration and resistance to apoptosis. Increased TWIST1 expression has consistently been associated with aggressive clinicopathological features in GC and has also been implicated in *Helicobacter pylori*-associated gastric carcinogenesis, suggesting that EMT-related changes may begin during the early stages of malignant transformation. Among structural biomarkers, the cadherin switch, characterized by loss of E-cadherin and gain of N-cadherin, represents one of the defining features of EMT. Increased N-cadherin expression weakens epithelial cell adhesion and enhances tumor cell interactions with the surrounding stroma, thereby facilitating invasion and dissemination. Likewise, vimentin, a canonical mesenchymal intermediate filament, is consistently upregulated during EMT and reflects the acquisition of a migratory phenotype rather than acting as an initiating event. Finally, β-catenin occupies a unique position linking cell adhesion with intracellular signaling. Following disruption of adherens junctions, β-catenin translocates to the nucleus, where activation of the WNT/β-catenin pathway promotes transcriptional programs involved in proliferation, stemness and invasion, thereby reinforcing the EMT phenotype.

Together, these additional biomarkers emphasize that EMT in GC is regulated by an interconnected molecular network rather than by a single pathway. While E-cadherin and SNAIL remain the most representative biomarkers of epithelial loss and transcriptional reprogramming, respectively, ZEB1, TWIST1, N-cadherin, vimentin and β-catenin provide complementary insights into the complexity and dynamic regulation of epithelial plasticity during GC progression [50,51,62,63].

## 6. Can the Cell Escape Immune Surveillance?

After acquiring stem cell-like properties and completing the epithelial–mesenchymal transition, tumor progression depends on another fundamental biological capability: the ability to evade immune destruction. Although GC frequently develops within a chronically inflamed microenvironment, malignant cells progressively acquire mechanisms that suppress antitumor immunity, allowing continued growth despite the presence of tumor-infiltrating immune cells. This concept has become increasingly relevant with the advent of immune checkpoint inhibitors, which have transformed the therapeutic landscape of advanced GC while simultaneously highlighting the importance of predictive immune biomarkers. Immune escape in GC is biologically heterogeneous. Some tumors evade immune surveillance through the expression of inhibitory checkpoint molecules that suppress cytotoxic T-cell activity, whereas others generate a highly immunogenic microenvironment as a consequence of deficient DNA mismatch repair or viral infection. Accordingly, molecular classification studies, particularly those proposed by The Cancer Genome Atlas (TCGA), have identified distinct immune-related GC subtypes, most notably Epstein–Barr virus (EBV)-positive and microsatellite instability-high (MSI-H) tumors, both characterized by prominent immune infiltration and increased expression of immune checkpoint molecules [56].

Among the numerous biomarkers investigated in this context, programmed death-ligand 1 (PD-L1) and mismatch repair deficiency/microsatellite instability (dMMR/MSI) have emerged as the two most clinically relevant. Together, they capture complementary aspects of tumor immune evasion: PD-L1 reflects adaptive immune suppression mediated through checkpoint signaling, whereas MSI/dMMR identifies tumors with increased neoantigen formation and heightened immunogenicity. Beyond their biological significance, both biomarkers have become integral components of routine pathological assessment because they directly guide the use of immune checkpoint inhibitors in patients with advanced GC [56,64].

### 6.1. PD-L1

Among the biomarkers associated with immune evasion in GC, programmed death-ligand 1 (PD-L1, CD274) has emerged as the most clinically influential. Physiologically, the interaction between PD-L1 expressed on tumor cells or immune cells and its receptor PD-1 on activated T lymphocytes serves as a regulatory mechanism that limits excessive immune activation and maintains peripheral immune tolerance. Malignant cells exploit this pathway by upregulating PD-L1, thereby attenuating cytotoxic T-cell activity and establishing an immunosuppressive tumor microenvironment that favours tumor survival and progression. Consequently, PD-L1 has evolved from a molecule involved in immune homeostasis into one of the most extensively investigated predictive biomarkers in solid tumors, including GC. The biological relevance of PD-L1 in GC became evident with the landmark molecular classification proposed by The Cancer Genome Atlas (TCGA) in 2014. By analyzing 295 primary gastric adenocarcinomas, TCGA identified the Epstein–Barr virus (EBV)-positive subtype as a distinct molecular entity characterized by recurrent amplification of CD274 (PD-L1) and PDCD1LG2 (PD-L2). This observation represented the first comprehensive demonstration that immune checkpoint activation constitutes an intrinsic biological feature of a subset of GCs rather than a secondary consequence of tumor progression. Together with the prominent immune-cell infiltration observed in EBV-positive tumors, these findings established PD-L1 as a defining component of the immune landscape of GC and provided the biological rationale for subsequent therapeutic targeting of the PD-1/PD-L1 axis [56].

The clinical significance of PD-L1 was subsequently established through the development of immune checkpoint inhibitors. The phase II KEYNOTE-059 trial represented the first pivotal study demonstrating meaningful and durable responses to pembrolizumab in previously treated patients with advanced gastric or gastro-esophageal junction adenocarcinoma. Importantly, clinical benefit was observed predominantly in tumors expressing PD-L1, introducing the Combined Positive Score (CPS) as a practical method for patient selection and establishing PD-L1 as a predictive biomarker for anti-PD-1 therapy. Unlike conventional tumor proportion scores used in other malignancies, CPS incorporates PD-L1 expression on both tumor cells and tumor-associated immune cells, reflecting the complex immune microenvironment characteristic of GC. Subsequent phase III trials refined the clinical application of PD-L1 by demonstrating that treatment benefit depends not simply on the presence of PD-L1 expression but also on its magnitude. In KEYNOTE-062, pembrolizumab-based therapy showed progressively greater benefit with increasing CPS values, supporting the concept that PD-L1 functions as a quantitative rather than binary biomarker. This study reinforced the clinical relevance of CPS assessment and highlighted the importance of standardized pathological evaluation for therapeutic decision-making [65].

The role of PD-L1 as a predictive biomarker was firmly established by the landmark CheckMate 649 trial, which transformed the first-line management of advanced HER2-negative GC. In this large international phase III study, the addition of nivolumab to chemotherapy significantly improved both overall survival and progression-free survival, particularly in patients with PD-L1 CPS ≥ 5. These findings led to the incorporation of PD-L1 CPS into international treatment guidelines and established chemo-immunotherapy as the standard first-line approach for appropriately selected patients. More recently, the global phase III KEYNOTE-859 trial independently confirmed these observations, demonstrating significant improvements in survival outcomes with pembrolizumab plus chemotherapy while further supporting the predictive value of increasing PD-L1 CPS. Together, these studies firmly established PD-L1 assessment as an important component of routine pathological evaluation in advanced GC. Although PD-L1 is now primarily regarded as a predictive biomarker for immunotherapy, its biological significance extends beyond treatment selection. PD-L1 expression is closely associated with an inflamed tumor microenvironment characterized by abundant tumor-infiltrating lymphocytes, interferon-γ signaling, and adaptive immune resistance. Moreover, increased PD-L1 expression is frequently observed in molecular subtypes with high immunogenicity, particularly EBV-positive and MSI-high GCs, reflecting the intimate relationship between genomic instability, host immune responses and immune checkpoint activation. These observations underscore that PD-L1 is not merely a therapeutic marker but also a surrogate of the dynamic interaction between tumor cells and the host immune system. Despite its central role in clinical practice, PD-L1 is an imperfect biomarker. Intratumoral heterogeneity, temporal variation in expression, differences between primary tumors and metastatic lesions, and variability among antibody clones and scoring methodologies can all influence PD-L1 assessment. Furthermore, objective responses to immune checkpoint inhibitors may occur in some patients with low or absent PD-L1 expression, whereas a proportion of PD-L1-positive tumors fail to respond. These limitations indicate that PD-L1 should not be interpreted in isolation but rather as one component of a broader immunological context integrating molecular subtype, tumor microenvironment and genomic features [56,66,67,68].

The increasing recognition of these limitations has shifted attention towards complementary biomarkers capable of capturing tumor immunogenicity through different biological mechanisms. Among these, mismatch repair deficiency (dMMR) and microsatellite instability (MSI) have emerged as the most robust companions to PD-L1 assessment, identifying tumors with high neoantigen burden and exceptional sensitivity to immune checkpoint blockade. Together, PD-L1 and MSI represent complementary biomarkers that define distinct, yet overlapping, mechanisms of immune responsiveness in GC [56,64]. The evolution of PD-L1 from a molecular feature of immune escape to an important predictive biomarker guiding immunotherapy in GC is summarized in the landmark biological studies and pivotal clinical trials presented in Table 6.

### 6.2. Mismatch Repair Deficiency (dMMR) and Microsatellite Instability (MSI)

While PD-L1 reflects an adaptive mechanism by which GC evades immune destruction, mismatch repair deficiency (dMMR) and microsatellite instability (MSI) define a fundamentally different biological phenomenon. Rather than describing how tumor cells suppress immune responses, MSI identifies a subgroup of GCs that become intrinsically immunogenic through the progressive accumulation of somatic mutations. As defective DNA mismatch repair generates an exceptionally high neoantigen burden, these tumors elicit robust immune-cell infiltration, activate interferon-driven inflammatory pathways, and ultimately become highly susceptible to immune checkpoint inhibition. Consequently, MSI has evolved from a molecular characteristic of genomic instability into one of the most influential biomarkers guiding prognostic assessment and therapeutic decision-making in GC. MSI-high GCs account for approximately 8–10% of gastric adenocarcinomas and exhibit clinicopathological characteristics that distinguish them from the other molecular subtypes of the disease. They occur more frequently in elderly patients, preferentially arise in the distal stomach, are commonly associated with the intestinal histological subtype, and frequently display prominent lymphoid infiltration together with medullary-like or poorly differentiated morphology. Unlike colorectal cancer, where hereditary Lynch syndrome represents an important cause of mismatch repair deficiency, the overwhelming majority of MSI-high GCs are sporadic and result from epigenetic silencing of the MLH1 promoter, whereas germline alterations account for only a small minority of cases. These observations indicate that MSI in GC primarily reflects a tumor-specific evolutionary pathway rather than an inherited cancer predisposition syndrome [56,70,71].

The modern understanding of MSI in GC was fundamentally reshaped by The Cancer Genome Atlas (TCGA), which identified MSI as one of the four major molecular subtypes of gastric adenocarcinoma. This landmark classification demonstrated that MSI-high tumors are characterized by extensive somatic hypermutation, recurrent alterations affecting multiple cancer-related signaling pathways, abundant tumor-infiltrating immune cells, and marked activation of immune-response genes. Importantly, TCGA established that MSI is not simply a consequence of defective DNA repair but represents a biologically distinct subtype with its own genomic architecture, tumor microenvironment, and clinical behavior. This molecular framework subsequently provided the biological rationale for the successful implementation of immune checkpoint blockade in GC [56].

Beyond its biological significance, MSI rapidly emerged as a clinically relevant biomarker. Initial evidence came from translational analyzes of the MAGIC trial, which demonstrated that patients with MSI-high or dMMR GCs experience significantly better survival than those with microsatellite-stable tumors despite deriving little or no benefit from perioperative chemotherapy. These findings challenged the traditional “one-size-fits-all” approach to perioperative treatment, suggesting that molecular subtype may be more informative than anatomical stage when selecting therapeutic strategies for resectable disease. The prognostic and predictive value of MSI was subsequently strengthened by the landmark individual patient data meta-analysis conducted by Pietrantonio et al., which integrated data from four major randomized clinical trials. By analyzing more than 1500 patients with resectable GC, the authors confirmed that MSI-high tumors consistently exhibit superior long-term survival while showing minimal benefit from perioperative or adjuvant chemotherapy. This study provided the highest level of evidence supporting the incorporation of MSI into clinical decision-making and firmly established the biomarker as an important determinant of treatment selection in localized GC. The therapeutic significance of MSI became even more evident with the introduction of immune checkpoint inhibitors. The hypermutated phenotype of MSI-high GCs generates a diverse repertoire of neoantigens capable of eliciting strong cytotoxic T-cell responses, thereby creating an immune microenvironment particularly susceptible to PD-1 blockade. Unlike many other biomarkers whose predictive value varies considerably across studies, the association between MSI and immunotherapy efficacy has remained remarkably consistent. A meta-analysis of randomized clinical trials, including KEYNOTE-061, KEYNOTE-062, CheckMate 649, and JAVELIN Gastric 100, demonstrated that MSI-high GCs derive substantially greater survival benefit from PD-1 inhibition than microsatellite-stable tumors, regardless of treatment regimen or study population. More recently, biomarker analyzes from KEYNOTE-859 further reinforced these observations, confirming that MSI-high tumors remain among the most immunotherapy-responsive molecular subgroups in the first-line treatment of advanced GC [67,69,70,71].

From a practical perspective, MSI also possesses several advantages over other predictive biomarkers currently used in GC. Whereas PD-L1 expression is influenced by intratumoral heterogeneity, dynamic regulation, and methodological variability in scoring systems, MSI represents a stable genomic alteration that can be reliably assessed either by immunohistochemical evaluation of MLH1, PMS2, MSH2, and MSH6 protein expression or by molecular microsatellite testing. The excellent concordance between these complementary approaches has facilitated the widespread adoption of MSI testing in routine pathology practice, where it has become an important component of the biomarker panel recommended for patients with advanced GC.

Although MSI-high tumors constitute only a relatively small fraction of gastric adenocarcinomas, their biological and clinical impact is disproportionately large. They represent a molecular subtype with distinctive pathological features, favorable intrinsic prognosis, reduced dependence on conventional cytotoxic chemotherapy, and exceptional sensitivity to immune checkpoint inhibition. Together with PD-L1, MSI captures a complementary dimension of tumor–immune biology in GC. While PD-L1 reflects adaptive immune suppression within an inflamed tumor microenvironment, MSI identifies tumors whose genomic instability generates the very immune response that checkpoint inhibitors subsequently unleash. The integration of these biomarkers therefore provides a more comprehensive framework for molecular classification, therapeutic stratification, and precision oncology in GC [56,64,69,70,71]. The progressive evolution of dMMR/MSI from a defining molecular subtype of GC to a clinically important biomarker guiding prognostic assessment, treatment selection, and immunotherapy is summarized in the landmark biological studies and pivotal clinical evidence presented in Table 6.

### 6.3. Additional Biomarkers

Although PD-L1 and MSI currently constitute the cornerstone biomarkers guiding immunotherapy in GC, several additional immune-related biomarkers have emerged that further refine patient stratification and provide complementary insights into tumor–immune interactions. While none has yet achieved the same level of clinical implementation, accumulating evidence suggests that these markers may contribute to future biomarker-driven therapeutic algorithms. Tumor mutational burden (TMB) has attracted considerable interest as a surrogate measure of tumor immunogenicity. High TMB reflects the accumulation of somatic mutations capable of generating neoantigens recognized by the host immune system. Although TMB frequently overlaps with MSI-high GCs, a subset of microsatellite-stable tumors also exhibits elevated mutational burden and may derive meaningful benefit from immune checkpoint inhibition. Nevertheless, the optimal cut-off values, sequencing platforms, and the substantial overlap with MSI have limited the routine clinical adoption of TMB as an independent biomarker in GC [64,72].

Another biologically distinct subgroup is represented by Epstein–Barr virus (EBV)-associated gastric carcinoma, which accounts for approximately 9% of GCs in the TCGA molecular classification. EBV-positive tumors are characterized by extensive immune-cell infiltration, recurrent amplification of the PD-L1 (CD274) and PD-L2 (PDCD1LG2) loci, and a highly inflamed tumor microenvironment. These features contribute to their remarkable sensitivity to immune checkpoint blockade, making EBV status an attractive biomarker for patient selection, although routine testing remains less widespread than PD-L1 or MSI assessment. Beyond the PD-1/PD-L1 axis, several additional immune checkpoint molecules have emerged as potential therapeutic targets. CTLA-4, LAG-3, and TIGIT participate in distinct mechanisms of T-cell exhaustion and immune suppression within the GC microenvironment. Their expression has been associated with increased immune infiltration and, in some studies, with favorable responses to combination immunotherapy. Early-phase clinical trials evaluating dual checkpoint blockade, particularly combinations targeting PD-1 together with CTLA-4 or LAG-3, have demonstrated encouraging activity, although these biomarkers have not yet been incorporated into routine clinical practice [56,64].

Collectively, these emerging biomarkers underscore the increasing complexity of immune regulation in GC. Rather than replacing PD-L1 or MSI, they are likely to complement existing biomarkers by identifying additional biological subsets and refining patient selection for future immunotherapeutic strategies. As comprehensive molecular profiling becomes more widely implemented, integrated biomarker panels incorporating genomic, viral, and immune parameters are expected to provide a more nuanced characterization of tumor–immune interactions and further advance precision immuno-oncology in GC [56,64].

## 7. Has the Tumor Become Dependent on Actionable Oncogenic Pathways?

The preceding biological questions explored the progressive transformation of normal gastric epithelial cells into malignant cells, from the preservation of lineage identity and acquisition of stem cell-like properties to epithelial plasticity and immune escape. Together, these biological processes explain how GC develops, adapts, and survives. However, from a clinical perspective, understanding tumor biology alone is insufficient. The final and perhaps most consequential question is whether tumor evolution has generated molecular vulnerabilities that can be therapeutically exploited. Unlike the biomarkers discussed in the previous sections, actionable biomarkers are not selected primarily because they reflect a biological phenotype but because they identify tumor-specific molecular dependencies. These alterations represent oncogenic pathways that become essential for tumor growth, survival, or therapeutic response, allowing the transition from descriptive tumor biology to precision oncology. Their clinical value therefore extends beyond diagnosis or prognosis, directly influencing treatment selection and increasingly defining the standard of care for patients with advanced GC [56,64].

Although comprehensive genomic studies have identified numerous molecular alterations in gastric adenocarcinoma, only a limited number have demonstrated sufficient biological relevance, reproducible pathological assessment, and consistent clinical benefit to become established therapeutic biomarkers. Among these, HER2 remains the archetype of successful targeted therapy in GC, fundamentally changing the management of advanced disease through HER2-directed monoclonal antibodies and antibody–drug conjugates. More recently, FGFR2b has emerged as a second clinically actionable target following the development of bemarituzumab, providing proof that additional molecular dependencies can be successfully translated into therapeutic opportunities. Several other alterations, including MET, EGFR, KRAS, PIK3CA, NTRK fusions, and angiogenic pathways involving VEGFR2, continue to expand the therapeutic landscape of GC. Although many remain investigational or are applicable only to selected molecular subsets, they illustrate the ongoing transition from single-biomarker testing toward comprehensive molecular profiling, where multiple actionable alterations may coexist within the same tumor. Accordingly, this final biological domain examines the biomarkers that currently define precision oncology in GC, highlighting how molecular characterization has evolved from improving our understanding of tumor biology to directly guiding individualized therapeutic decision-making [56,64,73].

### 7.1. Human Epidermal Growth Factor Receptor 2 (HER2)

Among the molecular alterations that have transformed the therapeutic landscape of GC, human epidermal growth factor receptor 2 (HER2) occupies a unique position as the first biomarker successfully translated into routine precision oncology. Unlike lineage-associated or prognostic biomarkers, the clinical significance of HER2 lies in its role as an oncogenic driver whose amplification creates a therapeutically exploitable molecular dependency. The recognition that a subset of gastric adenocarcinomas harbours HER2 amplification fundamentally changed the management of advanced disease, demonstrating for the first time that molecular profiling could directly guide treatment selection and improve patient survival. Consequently, HER2 has become the prototype predictive biomarker in GC, paving the way for the subsequent development of biomarker-driven therapeutic strategies. HER2 is encoded by the ERBB2 gene on chromosome 17q12 and belongs to the epidermal growth factor receptor family of transmembrane receptor tyrosine kinases. Unlike other members of the HER family, HER2 has no known ligand and exerts its biological activity primarily through homo- or heterodimerization, leading to activation of multiple downstream signaling pathways, including PI3K/AKT/mTOR and RAS/RAF/MEK/ERK. Persistent activation of these pathways promotes tumor-cell proliferation, survival, angiogenesis, invasion, and resistance to apoptosis. The biological relevance of HER2 in GC was first demonstrated by Tanner and colleagues, who identified HER2 amplification and protein overexpression in a distinct subset of gastric carcinomas and associated these alterations with more aggressive clinicopathological characteristics. This seminal study established HER2 as a biologically relevant molecular alteration and provided the rationale for investigating HER2-directed therapeutic strategies [74].

Before HER2-targeted therapy could be incorporated into clinical practice, reliable pathological assessment was essential. Unlike breast carcinoma, gastric adenocarcinomas frequently display incomplete basolateral membranous staining and pronounced intratumoral heterogeneity, rendering breast cancer scoring systems unsuitable. To address these differences, Hofmann and colleagues developed a GC-specific HER2 scoring algorithm, which subsequently became the foundation of international testing guidelines. This methodological advance enabled the landmark ToGA trial, published by Bang et al. in 2010, which demonstrated that the addition of trastuzumab to platinum–fluoropyrimidine chemotherapy significantly improved overall survival in patients with HER2-positive advanced gastric or gastro-esophageal junction adenocarcinoma [75]. ToGA represented a turning point in GC management, establishing HER2 as the first predictive biomarker incorporated into routine clinical practice and providing the first unequivocal evidence that precision oncology could improve outcomes in this disease. Subsequent molecular studies further refined the biological interpretation of HER2-positive GC. The Cancer Genome Atlas (TCGA) identified ERBB2 amplification as one of the defining molecular events of the chromosomal instability (CIN) subtype, explaining its preferential association with intestinal-type tumors and proximally located GCs. At the same time, increasing recognition of marked intratumoral and intertumoral HER2 heterogeneity revealed important biological and clinical challenges. Discordant HER2 expression between primary tumors and metastatic deposits, together with regional variation within individual tumors, contributes to diagnostic uncertainty and may partially explain both primary and acquired resistance to HER2-directed therapies. These findings emphasize the importance of adequate tissue sampling, standardized pathological assessment, and, whenever feasible, reassessment of HER2 status during disease progression [56,75,76].

The evolution of HER2-targeted therapy has also illustrated the complexity of precision oncology in GC. Several strategies designed to improve upon trastuzumab-based treatment failed to demonstrate significant survival advantages, including lapatinib in the LOGiC and TyTAN trials, dual HER2 blockade with pertuzumab in the JACOB study, and the first-generation antibody–drug conjugate trastuzumab emtansine (T-DM1) in the GATSBY trial. Collectively, these studies demonstrated that HER2 amplification alone is insufficient to predict therapeutic success and highlighted the biological consequences of tumor heterogeneity, dynamic HER2 expression, and alternative resistance mechanisms. In contrast, the next-generation antibody–drug conjugate trastuzumab deruxtecan substantially renewed interest in HER2-directed therapy. The DESTINY-Gastric01 trial demonstrated significant improvements in objective response rate and overall survival in patients previously treated with trastuzumab, establishing trastuzumab deruxtecan as a new therapeutic standard in later treatment lines and confirming the clinical value of antibody–drug conjugates in GC [68,77,78,79,80].

Recent advances have further challenged the traditional binary classification of HER2-positive and HER2-negative GC. Increasing attention has focused on HER2-low tumors, generally defined as those exhibiting an immunohistochemical score of IHC 1+ or IHC 2+/ISH-negative. Although these neoplasms have historically been considered HER2-negative, the development of highly potent antibody–drug conjugates has demonstrated that even relatively low levels of HER2 expression may constitute therapeutically exploitable targets. Exploratory analyzes from DESTINY-Gastric01 suggested clinically meaningful activity of trastuzumab deruxtecan, particularly in IHC 2+/ISH-negative tumors, whereas responses appeared less consistent in the IHC 1+ subgroup, underscoring the biological heterogeneity of HER2-low disease. Consequently, several ongoing clinical trials are evaluating HER2-directed therapies in this emerging population. By contrast, the recently proposed concept of HER2-ultralow expression remains investigational in GC, and its biological and therapeutic significance has yet to be established. These observations suggest that HER2 expression should increasingly be viewed as a biological continuum rather than a strictly dichotomous biomarker, reflecting the evolving capabilities of next-generation targeted therapies. The Phase III DESTINY-Gastric04 trial subsequently demonstrated that trastuzumab deruxtecan provides superior efficacy to ramucirumab plus paclitaxel in the second-line treatment of HER2-positive unresectable or metastatic gastric or gastroesophageal junction adenocarcinoma after progression on trastuzumab-containing first-line therapy. In 494 randomized patients, median overall survival was 14.7 months with trastuzumab deruxtecan versus 11.4 months with ramucirumab plus paclitaxel (HR 0.70, 95% CI 0.55–0.90; *p* = 0.004), with significantly improved progression-free survival and a higher confirmed objective response rate (44.3% vs. 29.1%). These findings establish trastuzumab deruxtecan as an important second-line treatment option for HER2-positive advanced GC after trastuzumab-based first-line therapy [8,81]. The landmark studies that have shaped the biological understanding and clinical implementation of HER2-targeted therapy in GC are summarized in Table 7.

### 7.2. Fibroblast Growth Factor Receptor 2b (FGFR2b)

Fibroblast growth factor receptor 2b (FGFR2b) has emerged as one of the most promising actionable biomarkers in GC, representing the next major advance in biomarker-driven therapy after HER2. While HER2 established the feasibility of precision oncology in GC, FGFR2b has expanded this paradigm by identifying a distinct molecular subgroup characterized by aberrant fibroblast growth factor signaling. The successful clinical development of FGFR2b-directed therapies further illustrates how molecular pathology has evolved from merely describing tumor biology to directly guiding therapeutic decision-making. FGFR2 is a member of the fibroblast growth factor receptor family of transmembrane receptor tyrosine kinases and plays an essential role in epithelial development, tissue homeostasis, and cellular differentiation. Alternative splicing generates two principal isoforms, FGFR2b and FGFR2c, of which FGFR2b is predominantly expressed in epithelial tissues, including the gastric mucosa. In gastric adenocarcinoma, aberrant activation of FGFR2 most commonly results from gene amplification accompanied by protein overexpression, leading to constitutive activation of downstream signaling pathways such as RAS/MAPK, PI3K/AKT, PLCγ, and STAT. These alterations promote tumor proliferation, invasion, angiogenesis, and metastatic dissemination, supporting the concept that FGFR2 functions as an oncogenic driver in a subset of GCs [82].

The biological significance of FGFR2 was initially demonstrated in preclinical studies showing that FGFR2-amplified GC cells exhibit oncogene dependency and that pharmacological inhibition of FGFR2 markedly suppresses tumor growth. Subsequent clinicopathological investigations confirmed that FGFR2 amplification and overexpression are associated with aggressive tumor behavior, diffuse histology, advanced-stage disease, and unfavorable prognosis. However, later genomic analyzes also revealed substantial intratumoral heterogeneity, demonstrating that FGFR2 amplification may be regionally distributed within individual tumors and may differ between primary and metastatic lesions. These observations highlighted important challenges for biomarker assessment and emphasized that accurate patient selection depends not only on detecting FGFR2 alterations but also on accounting for spatial heterogeneity. Unlike HER2, where therapeutic eligibility primarily relies on gene amplification and protein overexpression, the clinical development of FGFR2-directed therapy has progressively shifted the focus towards FGFR2b protein expression as the most relevant predictive biomarker. This transition reflects the mechanism of action of bemarituzumab, a first-in-class monoclonal antibody specifically targeting the extracellular FGFR2b isoform expressed on the tumor-cell membrane. Consequently, immunohistochemical assessment has become central to patient selection, illustrating how the biological characteristics of a therapeutic target directly influence biomarker development [82,83,84,85].

The clinical relevance of FGFR2b was established through the FIGHT program, which provided the first convincing evidence that selective FGFR2b inhibition improves clinical outcomes in advanced GC. In the randomized Phase II FIGHT trial, the addition of bemarituzumab to mFOLFOX chemotherapy resulted in clinically meaningful improvements in progression-free survival, overall survival, and objective response rate compared with chemotherapy alone in patients with HER2-negative, FGFR2b-positive advanced gastric or gastro-oesophageal junction adenocarcinoma. Importantly, the final analysis demonstrated that the greatest therapeutic benefit was achieved in tumors exhibiting FGFR2b expression in at least 10% of tumor cells, reinforcing the concept that biomarker quantification, rather than simple positivity, is critical for optimal patient selection. These findings established FGFR2b as a clinically actionable biomarker and highlighted the importance of integrating quantitative immunohistochemical evaluation into therapeutic decision-making. The ongoing Phase III FORTITUDE-101 trial is expected to determine whether bemarituzumab combined with chemotherapy and immunotherapy can become a new first-line standard of care for patients with FGFR2b-overexpressing GC. Beyond bemarituzumab, several additional FGFR-directed therapeutic strategies, including selective tyrosine kinase inhibitors, antibody–drug conjugates, and bispecific antibodies, are currently under clinical investigation, reflecting the rapid evolution of this therapeutic field. Collectively, these developments position FGFR2b as one of the most compelling emerging biomarkers in GC and further reinforce the concept that precision oncology increasingly depends on integrating molecular alterations with robust pathological assessment [86,87,88]. The landmark studies supporting the biological rationale and clinical implementation of FGFR2b-targeted therapy are summarized in Table 7.

### 7.3. Emerging Actionable Biomarkers: Broadening the Landscape of Precision Oncology

Although HER2 and FGFR2b currently represent the most mature predictive biomarkers for targeted therapy in GC, advances in molecular profiling have identified an expanding spectrum of additional oncogenic alterations that may further extend the precision oncology landscape. Unlike HER2 and FGFR2b, however, most of these molecular targets have not yet achieved routine clinical implementation because their biological complexity, low prevalence, intratumoral heterogeneity, or inconsistent predictive performance have limited successful therapeutic translation. Consequently, these biomarkers are best regarded as emerging rather than fully established therapeutic targets. Among receptor tyrosine kinases, MET has received considerable attention because amplification of the MET proto-oncogene results in constitutive activation of signaling pathways regulating proliferation, invasion, epithelial–mesenchymal transition, angiogenesis, and metastatic dissemination. Initial clinical trials evaluating MET-directed therapies produced largely disappointing results despite encouraging preclinical evidence. Subsequent analyzes suggested that these failures were primarily attributable to heterogeneous patient selection, variable biomarker definitions, and the inclusion of tumors with MET overexpression rather than true gene amplification. More recent studies employing stricter molecular selection criteria have demonstrated encouraging clinical activity, indicating that the therapeutic potential of MET inhibition depends critically on precise biomarker assessment rather than on MET expression alone [56,85,89].

A similar developmental trajectory has been observed for EGFR, another receptor tyrosine kinase frequently overexpressed in gastric adenocarcinoma. Despite its established oncogenic role in several epithelial malignancies, randomized trials investigating anti-EGFR monoclonal antibodies failed to demonstrate meaningful survival benefits in unselected GC populations. These findings highlighted an important principle that has subsequently influenced biomarker development across precision oncology: biological expression alone does not necessarily confer therapeutic vulnerability. Instead, successful targeted therapy requires robust predictive biomarkers capable of identifying tumors that remain genuinely dependent on the corresponding oncogenic pathway. Beyond receptor tyrosine kinases, increasing attention has focused on alterations involving intracellular signaling pathways. Activating PIK3CA mutations and dysregulation of the PI3K/AKT/mTOR pathway contribute to tumor progression, metabolic adaptation, and resistance to systemic therapy, while KRAS alterations may influence responsiveness to receptor-directed treatment and represent potential targets for recently developed allele-specific inhibitors. Although these alterations occur in only a subset of GCs, they illustrate the growing recognition that therapeutic vulnerabilities extend beyond membrane receptors to encompass downstream signaling networks that sustain malignant growth [56,90,91].

Nicotinamide N-methyltransferase (NNMT) has also emerged as a potential biomarker and therapeutic target in gastric cancer. NNMT consumes S-adenosylmethionine (SAM) and influences cellular methylation capacity and NAD metabolism, thereby contributing to metabolic and epigenetic regulation. Increased NNMT expression has been implicated in gastric cancer progression and treatment resistance, while experimental NNMT inhibition has shown therapeutic potential, including in the context of chemoresistance [92]. The metabolic and epigenetic effects of NNMT-mediated SAM consumption have also been characterized, supporting its potential role as a link between cellular metabolism and epigenetic regulation [93].

Additional genomic alterations, including BRAF V600E mutations, RET rearrangements, and NTRK gene fusions, are individually uncommon but have acquired increasing clinical relevance following the development of tumor-agnostic targeted therapies. Unlike conventional biomarker strategies that were developed specifically for GC, these alterations are identified through comprehensive molecular profiling across multiple tumor types and permit the use of highly selective targeted agents irrespective of tissue of origin. Their clinical significance therefore extends beyond prevalence alone and reflects the broader transition toward genomically driven oncology. Collectively, these emerging biomarkers illustrate that the future of precision oncology in GC will not depend on the discovery of a single dominant therapeutic target but rather on the simultaneous identification of multiple, often low-frequency, actionable molecular alterations. As next-generation sequencing becomes increasingly integrated into routine pathological practice, comprehensive molecular profiling is expected to complement immunohistochemical biomarker assessment, enabling more refined patient stratification and facilitating individualized therapeutic decision-making. Rather than replacing established biomarkers such as HER2 or FGFR2b, these emerging molecular alterations are likely to expand the therapeutic armamentarium available for patients with GC, further reinforcing the central role of molecular pathology in precision medicine [94,95,96]. In addition, serum biomarkers such as AFP and CEA, particularly when integrated with imaging characteristics, may contribute to the differential identification of gastric adenocarcinoma, illustrating the potential value of combining biomarker and phenotypic information in histologically distinct GC subtypes [97]. Emerging approaches, including PD-L1-specific near-infrared fluorescence/Cerenkov luminescence imaging, further illustrate the potential expansion of biomarker assessment beyond conventional tissue-based methods, although such approaches remain investigational [98].

**Figure 2:**
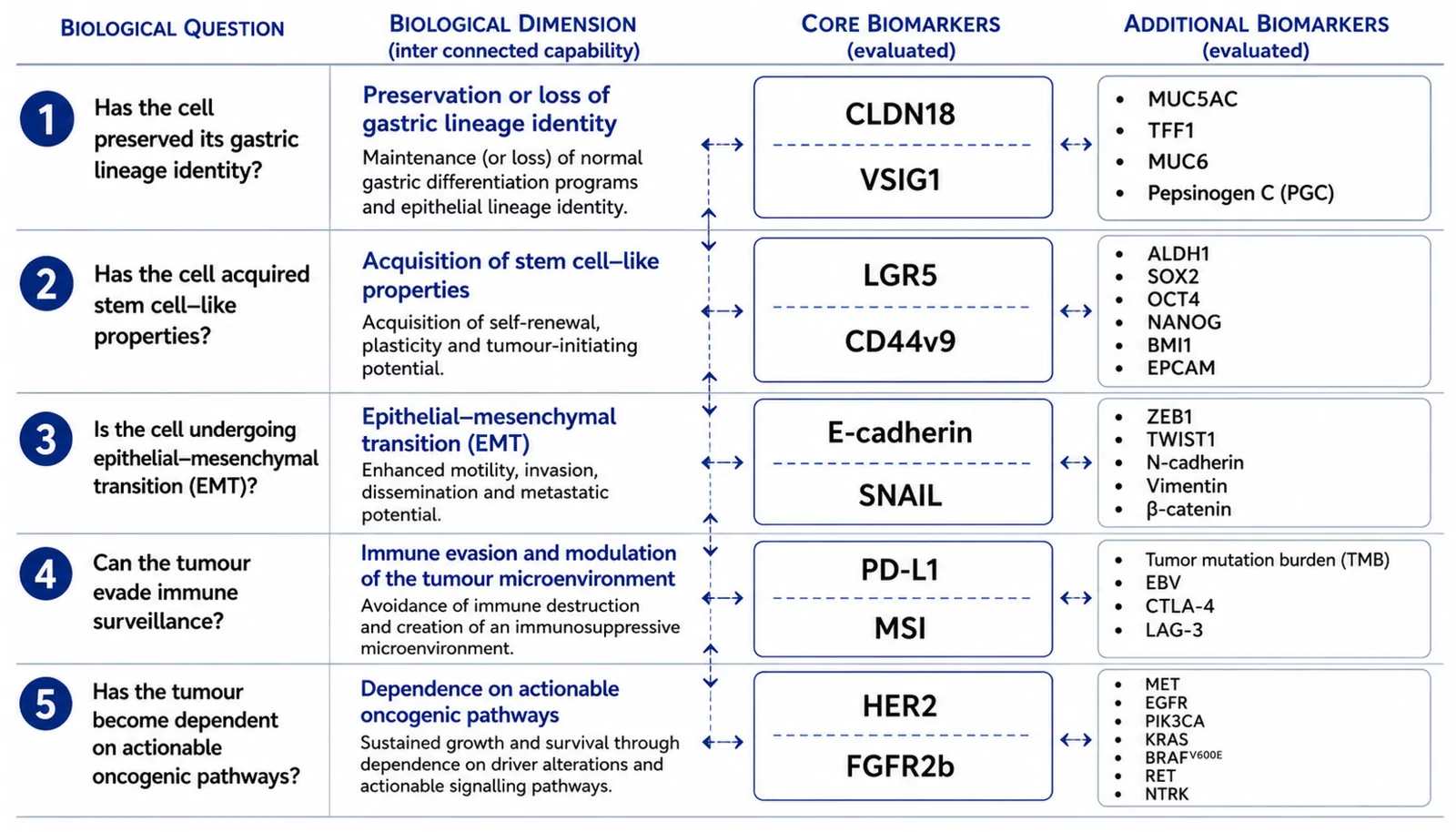
Biology-oriented conceptual framework summarizing five interconnected biological questions used to organize biomarker evaluation in gastric carcinogenesis. Core and complementary biomarkers discussed within each biological domain are shown.

The biology-oriented framework proposed in this review is recapitulated in Figure 2, integrating five interconnected biological questions together with the core and complementary biomarkers discussed within each biological domain.

### 7.4. Practical Biomarker Testing Strategy in Gastric Cancer

In clinical practice, biomarker testing in GC should be approached as an integrated assessment rather than as an isolated evaluation of individual markers. At the diagnosis of unresectable or metastatic disease, assessment of HER2, PD-L1, MSI/dMMR, and CLDN18.2 provides a core biomarker profile for treatment stratification, while FGFR2b and broader molecular profiling may provide additional information in selected clinical contexts. The practical characteristics, interpretation criteria, clinical roles, and current levels of evidence for these and other relevant biomarkers are summarized in Table 8.

Biomarker assessment should be performed on representative, adequately cellular tumor tissue, with specimen selection adapted to the biomarker and clinical context. Both endoscopic biopsy and resection specimens can be suitable for initial testing, whereas metastatic tissue may be assessed when clinically relevant or when the primary tumor is unavailable. For biomarkers characterized by substantial intratumoral heterogeneity, particularly HER2 and CLDN18.2, multiple tumor fragments or blocks should be evaluated when feasible to reduce sampling-related false-negative results. For HER2 assessment, current recommendations favor obtaining multiple endoscopic biopsy fragments when advanced disease is diagnosed, while resection material should be sampled from morphologically representative tumor areas. PD-L1 assessment requires adequate viable tumor and associated immune-cell populations, and results should be interpreted with awareness of regional variation in expression. When tissue is limited, biomarker testing should be prioritized according to the clinical scenario, while preserving sufficient material for complementary molecular testing. Testing of a metastatic lesion may be particularly informative when biomarker discordance between primary and metastatic disease is suspected or when the metastatic specimen provides substantially better-quality tissue than the primary tumor.

Interpretation should account for assay-specific scoring systems and positivity thresholds rather than treating biomarker expression as a binary and interchangeable parameter. This is particularly relevant for HER2, PD-L1, CLDN18.2, and FGFR2b, for which assay characteristics, scoring criteria, and clinically relevant thresholds differ substantially. Biomarker results should therefore be interpreted together with specimen adequacy, tumor heterogeneity, and the clinical context. When results are discordant between specimens or when tumor evolution is suspected, reassessment on an alternative or newly obtained specimen may be considered when the result is expected to influence subsequent clinical decision-making.

**Figure 3:**
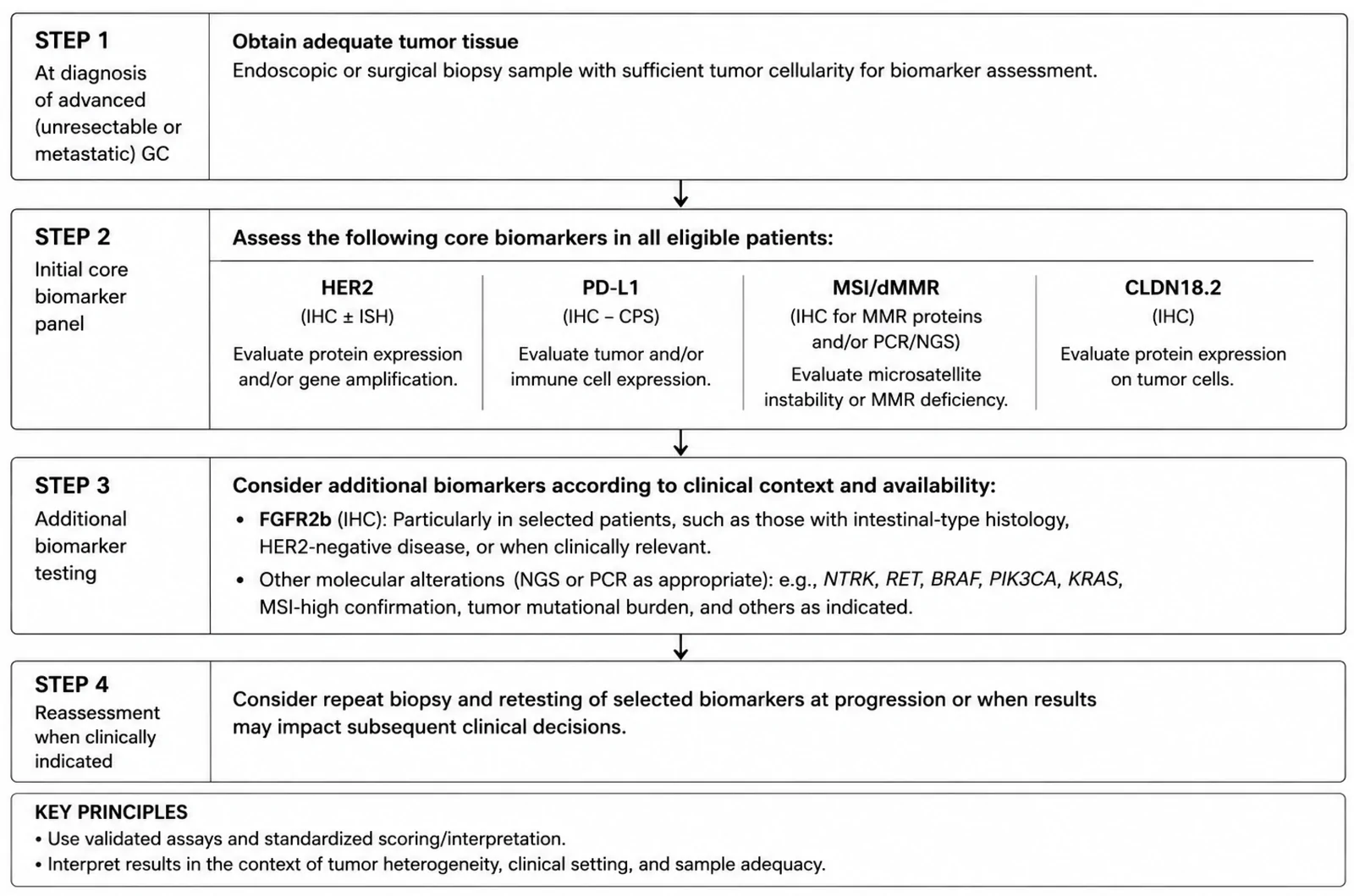
Workflow for biomarker testing in gastric cancer. Stepwise approach to initial and context-dependent biomarker assessment.

The proposed testing workflow is summarized in Figure 3, which illustrates the progression from initial tissue assessment and core biomarker testing to context-dependent molecular testing and selected reassessment during disease evolution. Biomarkers such as VSIG1, LGR5, CD44v9, and NNMT currently remain primarily investigational and should therefore be interpreted as biologically informative or emerging research biomarkers rather than as substitutes for clinically established biomarkers in routine treatment selection.

**Figure 4:**
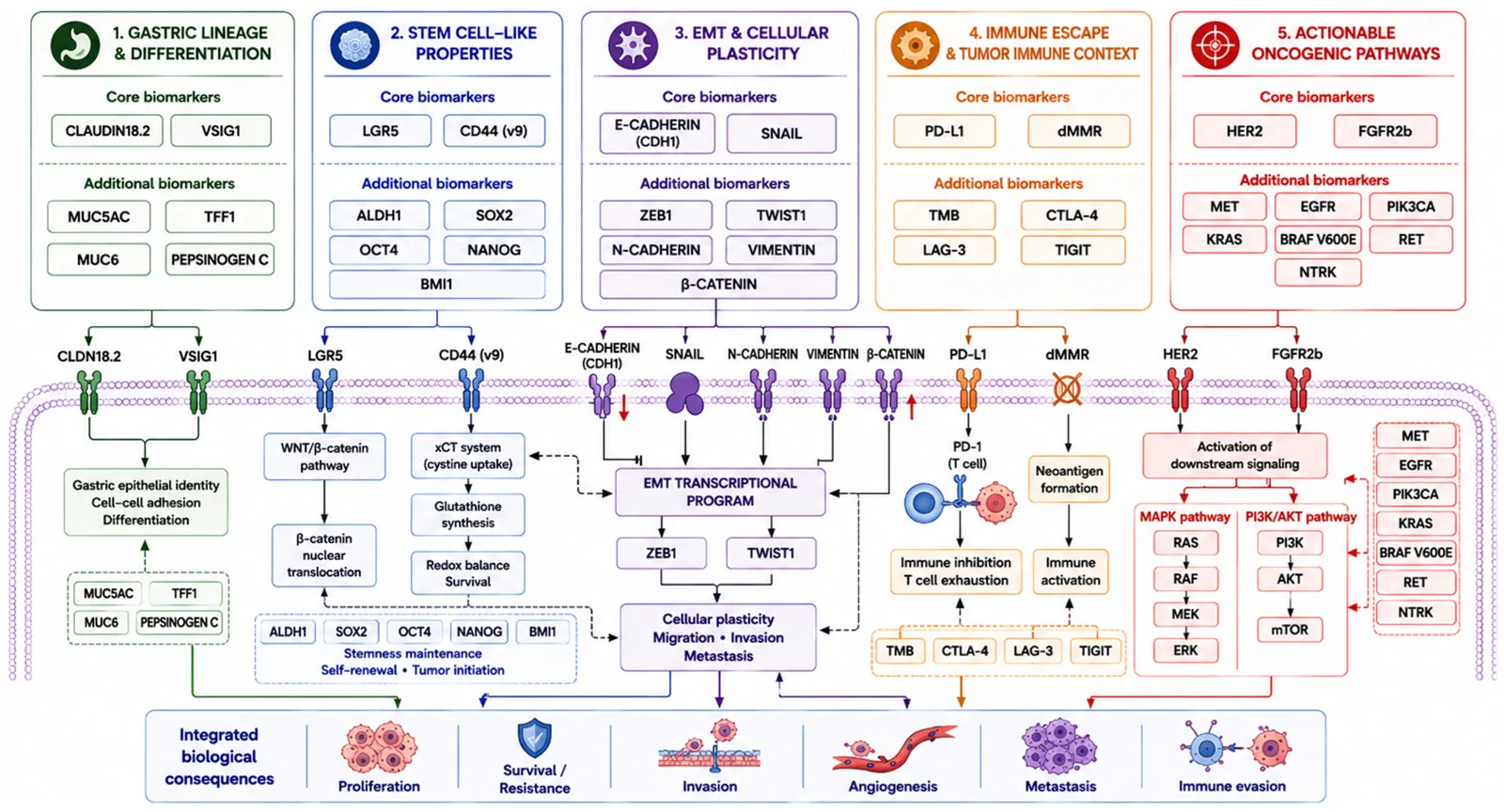
Major signaling pathways associated with gastric cancer biomarkers. Schematic overview of the principal signaling pathways linking the five biological domains and their core and additional biomarkers to gastric cancer-related cellular processes and phenotypic consequences.

The principal signaling pathways associated with the five biological domains and their respective biomarkers are summarized in Figure 4.

## 8. Conclusions

The expanding landscape of GC biomarkers reflects far more than the accumulation of individual molecular discoveries. Rather, it mirrors the progressive biological evolution of gastric epithelial cells during carcinogenesis, from preservation of lineage identity to the acquisition of stem cell-like properties, epithelial plasticity, immune escape, and ultimately therapeutically actionable molecular dependencies. Understanding biomarkers within this evolutionary context provides a more coherent interpretation of tumor biology than considering each marker as an isolated molecular entity. In this review, we adopted a biology-oriented framework that organized the available evidence around five fundamental biological questions representing the major biological transitions occurring during GC progression. This approach allowed biomarkers with related biological functions to be evaluated comparatively, facilitating the identification of those most consistently supported by current evidence while simultaneously acknowledging the complementary contribution of additional markers. Rather than proposing a new molecular classification, this framework offers a conceptual strategy for integrating diverse biomarkers into a biologically meaningful model of gastric carcinogenesis.

The rapid expansion of precision oncology further highlights the evolving role of biomarkers in GC. Molecules initially investigated as indicators of tumor differentiation or biological behavior have increasingly become predictive biomarkers that directly influence therapeutic decision-making. Consequently, contemporary pathological assessment extends beyond diagnosis and prognostication to encompass patient stratification for targeted therapies and immunotherapy. As comprehensive molecular profiling, multiplex biomarker assessment, and next-generation sequencing become progressively integrated into routine clinical practice, future biomarker strategies will likely rely on the combined interpretation of multiple complementary biological domains rather than on individual biomarkers alone.

Although many candidate biomarkers continue to require prospective validation and methodological standardization before widespread clinical implementation, the biological framework presented here provides a structured approach for interpreting the rapidly expanding literature. By integrating molecular pathology with the interconnected biological dimensions of GC, this review aims to facilitate a more intuitive understanding of biomarker significance, support the development of rational multimarker panels, and strengthen the connection between tumor biology, diagnostic pathology, and precision oncology. Ultimately, viewing biomarkers as indicators of interconnected biological capabilities and tumor states rather than isolated molecular alterations may contribute to more biologically informed research and increasingly individualized management of patients with GC.

## Figures and Tables

**Table 1:** Biological questions used to organize evidence synthesis.

Biological Question	Biological Process Evaluated	Purpose
Has the cell preserved its gastric lineage identity?	Gastric differentiation and lineage commitment	Identify biomarkers reflecting preservation or loss of gastric epithelial identity
Has the cell acquired stem cell-like properties?	Stemness and cellular plasticity	Identify biomarkers associated with tumor plasticity and stem cell-like features
Is the cell undergoing epithelial–mesenchymal transition?	Epithelial–mesenchymal transition (EMT)	Evaluate biomarkers reflecting epithelial plasticity, loss of epithelial integrity, and acquisition of invasive potential
Can the tumor evade host immune surveillance?	Tumor–immune interaction	Evaluate biomarkers associated with immune escape mechanisms
Has the tumor become dependent on actionable oncogenic pathways?	Targetable oncogenic signaling and therapeutic vulnerabilities	Identify predictive biomarkers that guide targeted therapy and precision oncology

**Table 2:** Predefined criteria for biomarker evaluation and selection.

Criterion	Definition
Biological specificity	Evidence supporting a direct role within the biological domain under investigation
Human evidence	Validation in gastric adenocarcinoma cohorts
Evidence consistency	Reproducible findings across independent studies
Clinical relevance	Association with diagnosis, prognosis, prediction or therapy
Technical feasibility	Assessment using established pathological or molecular techniques

**Table 3:** (**A**) Landmark studies underpinning the selection of CLDN18.2 as a core biomarker of preserved gastric lineage identity. (**B**) Landmark studies underpinning the selection of VSIG1 as a core biomarker of preserved gastric lineage identity.

**(A) Landmark Studies Underpinning the Selection of CLDN18.2 as a Core Biomarker of Preserved Gastric Lineage Identity**				
**Reference**	**Study Design**	**Cohort**	**Principal Finding**	**Biological Milestone**
Sahin et al., 2008 [11]	Discovery/translational study	Experimental models and human gastric tissues	Identified CLDN18.2 as a gastric-restricted tight-junction protein with highly selective physiological expression.	Established the biological identity of CLDN18.
Dottermusch et al., 2019 [12]	Whole-slide immunohistochemical cohort	481 gastric adenocarcinomas	Comprehensively characterized CLDN18.2 prevalence, expression patterns, and marked intratumoral heterogeneity.	Validated pathological assessment of CLDN18.
Kwak et al., 2024 [14]	Multicentre clinicopathological study	1000 gastric adenocarcinomas (stage II–IV)	Defined CLDN18.2 prevalence and analyzed associations with HER2, FGFR2, PD-L1, MSI, and EBV status.	Expanded the molecular and clinicopathological landscape of CLDN18.
Pellino et al., 2021 [15]	Multicentre molecular pathology study	350 oesophagogastric adenocarcinomas	Evaluated CLDN18.2 prevalence together with molecular characteristics and clinically relevant scoring criteria.	Integrated CLDN18 into molecular pathology.
FAST Trial (Sahin et al., 2021) [16]	Phase II randomised clinical trial	Patients with advanced CLDN18.2-positive gastric/GEJ adenocarcinoma	First prospective demonstration that zolbetuximab improved clinical outcomes in CLDN18.2-positive disease.	Established predictive clinical utility.
Kubota et al., 2023 [19]	Contemporary clinicopathological validation	408 gastric/GEJ adenocarcinomas	Validated CLDN18.2 prevalence using the VENTANA companion diagnostic assay and contemporary scoring criteria.	Supported standardized biomarker assessment.
SPOTLIGHT (Shitara et al., 2023) [17]	Global Phase III randomised trial	565 patients with HER2-negative advanced gastric/GEJ adenocarcinoma	Demonstrated significant improvements in progression-free and overall survival with zolbetuximab plus mFOLFOX6.	Changed clinical practice.
GLOW (Shah et al., 2023) [18]	Global Phase III randomised trial	507 patients with HER2-negative advanced gastric/GEJ adenocarcinoma	Independently confirmed the efficacy of zolbetuximab combined with CAPOX.	Provided independent Phase III validation.
**(B) Landmark Studies Underpinning the Selection of VSIG1 as a Core Biomarker of Preserved Gastric Lineage Identity**				
**Reference**	**Study Design**	**Cohort**	**Principal Finding**	**Biological Milestone**
Scanlan et al., 2006 [20]	Gene discovery study	Human tissue expression profiling	First identified VSIG1 as a tissue-restricted immunoglobulin superfamily protein with predominant gastric epithelial expression.	Discovery of VSIG1 as a gastric lineage-associated molecule.
Oidovsambuu et al., 2011 [21]	Experimental functional study	Murine gastric epithelium	Demonstrated that VSIG1 is required for normal gastric epithelial differentiation and glandular organization.	Established the biological role of VSIG1 in gastric epithelial differentiation.
Chen et al., 2012 [22]	Clinicopathological study	232 gastric adenocarcinomas	Demonstrated reduced VSIG1 expression in GC and identified loss of expression as an adverse prognostic factor.	First evidence linking VSIG1 loss to tumor progression and poor prognosis.
Inoue et al., 2017 [23]	Functional and prognostic study	362 GCs and experimental models	Demonstrated tumor suppressor-like activity and identified preserved VSIG1 expression as an independent favorable prognostic factor.	Defined the functional significance of VSIG1 in GC.
Satala et al., 2022 [9]	Translational pathology study	94 gastric adenocarcinomas	Correlated VSIG1 expression with epithelial–mesenchymal transition, tumor stage and lymph-node metastasis, highlighting marked intratumoral heterogeneity.	Expanded the pathological interpretation of VSIG1 during tumor progression.
Satala et al., 2023 [24]	Clinicopathological study	60 gastric adenocarcinomas	Integrated VSIG1 with gastric and intestinal mucin phenotypes, demonstrating that VSIG1 reflects preservation of the gastric differentiation program rather than simple anatomical origin.	Positioned VSIG1 as a marker of gastric-enriched epithelial differentiation.

**Table 4:** (**A**) Landmark studies underpinning the selection of LGR5 as a core biomarker of stem cell-like properties in GC. (**B**) Landmark studies underpinning the selection of CD44v9 as a core biomarker of stem cell-like properties in GC.

**(A) Landmark Studies Underpinning the Selection of LGR5 as a Core Biomarker of Stem Cell-like Properties in GC**				
**Reference**	**Study Design**	**Cohort**	**Principal Finding**	**Biological Milestone**
Barker et al., 2010 [34]	Experimental lineage-tracing study	Mouse stomach and gastric organoid model	Identified LGR5 as the first bona fide adult gastric stem-cell marker and demonstrated its capacity for long-term self-renewal and multipotency.	Established LGR5 as the well-established marker of normal gastric stem cells.
Simon et al., 2012 [37]	Translational clinicopathological study	Gastrointestinal tumors, including gastric adenocarcinomas	Demonstrated increased LGR5 expression during gastric carcinogenesis and confirmed its association with stem cell-related molecular programs in human GC.	Provided one of the earliest clinicopathological validations of LGR5 in human GC.
Leushacke et al., 2016 [35]	Experimental stem-cell dynamics	Mouse pyloric epithelium	Demonstrated that LGR5^+^ gastric stem cells maintain epithelial homeostasis through symmetric division and neutral drift.	Defined the biological behavior of LGR5-positive gastric stem cells during tissue homeostasis.
Jang et al., 2013 [39]	Human tissue-based translational study	Normal gastric mucosa, intestinal metaplasia, gastric adenomas and early gastric carcinoma	Demonstrated expansion of LGR5-positive cells during intestinal metaplasia and early gastric neoplasia, supporting their involvement in early gastric carcinogenesis.	Linked LGR5-positive stem-cell expansion with early stages of human gastric tumorigenesis.
Zheng et al., 2013 [40]	Clinicopathological study	Gastric adenocarcinomas and precursor lesions	Demonstrated progressive LGR5 expression across the gastric carcinogenesis sequence and its association with adverse clinicopathological features.	Validated the clinicopathological relevance of LGR5 during GC progression.
Li et al., 2016 [36]	Genetic lineage-tracing mouse model	Conditional mouse model of gastric carcinogenesis	Demonstrated that LGR5-positive gastric stem cells can function as the cell of origin for invasive intestinal-type gastric adenocarcinoma.	Provided direct experimental evidence that LGR5-positive stem cells can initiate GC.
**(B) Landmark Studies Underpinning the Selection of CD44v9 as a Core Biomarker of Stem Cell-like Properties in GC**				
**Reference**	**Study Design**	**Cohort**	**Principal Finding**	**Biological Milestone**
Takaishi et al., 2009 [41]	Experimental and translational study	Human GC cell lines and primary GCs	Identified CD44-positive cells as tumor-initiating GC stem cells capable of self-renewal and tumor formation.	Foundation of the GC stem-cell concept.
Ishimoto et al., 2011 [42]	Mechanistic experimental study	Experimental cancer models	Demonstrated that CD44 variant isoforms stabilize xCT, suppress reactive oxygen species accumulation and promote survival of stem-like cancer cells.	Discovery of the CD44 variant–xCT–redox resistance pathway.
Hirata et al., 2013 [43]	Clinicopathological study	88 patients with early GC treated by ESD	Demonstrated that CD44v9 expression predicts metachronous recurrence after curative endoscopic resection.	First clinical validation of CD44v9 as a recurrence biomarker in GC.
Go et al., 2016 [44]	Large clinicopathological study	333 GCs	Demonstrated progressive CD44v9 expression during gastric carcinogenesis and established its prognostic significance in early GC.	Large-scale clinicopathological validation of CD44v9.
Kodama et al., 2017 [45]	Clinicopathological study	123 resected GCs	Demonstrated that CD44-positive stem-like cells, including CD44v9-positive populations, at the invasive tumor front are associated with poor survival.	Established the biological significance of stem-like cells at the invasive front.
Jogo et al., 2021 [46]	Translational study	GC patients and cell-line models	Demonstrated that CD44v9 promotes chemoresistance through regulation of intracellular ROS and independently predicts poor clinical outcome.	Functional validation of CD44v9-mediated therapy resistance.

**Table 5:** (**A**) Landmark studies underpinning the selection of E-cadherin as a core biomarker of epithelial–mesenchymal transition in GC. (**B**) Landmark studies underpinning the selection of SNAIL as a core biomarker of epithelial–mesenchymal transition in GC.

**(A) Landmark Studies Underpinning the Selection of E-Cadherin as a Core Biomarker of Epithelial–Mesenchymal Transition in GC**				
**Reference**	**Study Design**	**Cohort**	**Principal Finding**	**Biological Milestone**
Becker et al., 1994 [52]	Molecular and immunohistochemical analysis	53 gastric carcinomas	First demonstration of somatic CDH1 mutations predominantly in diffuse-type gastric carcinoma, linking E-cadherin dysfunction to loss of epithelial cohesion.	Established CDH1 as a tumor suppressor gene involved in diffuse gastric carcinogenesis.
Machado et al., 1998 [54]	Immunohistochemical clinicopathological study	50 gastric carcinomas	Abnormal E-cadherin expression correlated with diffuse histology, lymph node metastasis, advanced stage, and aggressive tumor behavior.	First clinicopathological validation of E-cadherin loss as a marker of tumor aggressiveness.
Machado et al., 2001 [53]	Molecular epigenetic study	Sporadic diffuse gastric carcinomas	Demonstrated promoter hypermethylation as a major mechanism of CDH1 inactivation in sporadic GC.	Established epigenetic silencing as a key mechanism of E-cadherin loss.
Corso et al., 2013 [55]	Integrated molecular and clinicopathological analysis	246 GCs	Combined assessment of CDH1 mutations, deletions, and protein expression demonstrated significant prognostic value of CDH1 alterations.	Validated the prognostic significance of CDH1 alterations in GC.
The Cancer Genome Atlas (TCGA) Research Network, 2014 [56]	Comprehensive genomic characterization	295 primary gastric adenocarcinomas	Identified frequent CDH1 alterations within the genomically stable molecular subtype, linking E-cadherin dysfunction to diffuse GC biology.	Integrated CDH1 into the molecular classification of gastric adenocarcinoma.
Figueiredo et al., 2022 [57]	Functional and mechanistic experimental study	Cell models and GC experimental systems	Demonstrated that E-cadherin dysfunction promotes abnormal cell–matrix interactions and invasive behavior through integrin β1 signaling.	Provided contemporary mechanistic evidence linking E-cadherin dysfunction to tumor invasion and EMT-associated plasticity.
**(B) Landmark Studies Underpinning the Selection of SNAIL as a Core Biomarker of Epithelial–Mesenchymal Transition in GC**				
**Reference**	**Study Design**	**Cohort**	**Principal Finding**	**Biological Milestone**
Rosivatz et al., 2002 [58]	Quantitative RT-PCR and molecular analysis	48 primary gastric carcinomas (28 diffuse, 20 intestinal) with matched non-neoplastic tissues	First study to investigate EMT regulators in GC, demonstrating that increased SNAIL expression is associated with reduced E-cadherin expression in diffuse-type gastric carcinoma.	First evidence establishing SNAIL as a principal transcriptional regulator of EMT in GC.
Rosivatz et al., 2006 [59]	Immunohistochemical study	Upper gastrointestinal adenocarcinomas (including gastric carcinomas)	Demonstrated that nuclear localization of SNAIL, rather than expression alone, is associated with E-cadherin repression and tumor progression.	Supported nuclear SNAIL expression as the biologically active form linked to EMT.
He et al., 2012 [60]	Clinicopathological and functional study	103 gastric carcinomas, 10 normal gastric tissues, and GC cell lines	Showed that SNAIL promotes dedifferentiation, stem cell-like phenotype, migration and invasion, and independently predicts poor overall survival.	Supported SNAIL as an independent prognostic biomarker and functional driver of GC progression.
Shin et al., 2012 [61]	Immunohistochemistry, tissue microarray, functional assays and gene-expression profiling	314 gastric adenocarcinomas	Demonstrated that SNAIL overexpression correlates with lymph node metastasis, lymphovascular invasion, advanced stage and poor prognosis, while functionally enhancing tumor invasiveness.	Large-scale clinicopathological and functional validation of SNAIL as a marker of aggressive GC.
Wu et al., 2024 [62]	Immunohistochemical prognostic study	190 patients with advanced GC	Confirmed that SNAIL overexpression in primary tumors and tumor deposits is associated with lymph node metastasis, tumor deposits and shorter disease-free survival.	Contemporary clinical validation of SNAIL as an EMT-associated biomarker in advanced GC.

**Table 6:** (**A**) Landmark studies underpinning the selection of PD-L1 as a core biomarker of immune evasion in GC. (**B**) Landmark studies underpinning the selection of dMMR/MSI as a core biomarker of immune responsiveness in GC.

**(A) Landmark Studies Underpinning the Selection of PD-L1 as a Core Biomarker of Immune Evasion in GC**				
**Reference**	**Study Design**	**Cohort**	**Principal Finding**	**Biological/Clinical Milestone**
The Cancer Genome Atlas (TCGA) Research Network, 2014 [56]	Integrated genomic and molecular analysis	295 primary gastric adenocarcinomas	Identified the EBV-positive molecular subtype characterized by recurrent CD274 (PD-L1) and PDCD1LG2 (PD-L2) amplification, establishing immune checkpoint activation as a defining biological feature of a subset of GCs.	Established the molecular basis of PD-L1 activation in GC and integrated it into the TCGA molecular classification.
KEYNOTE-059 (Fuchs et al., 2018) [65]	Phase II clinical trial	259 patients with previously treated advanced gastric/GEJ adenocarcinoma	Demonstrated durable responses to pembrolizumab, particularly in PD-L1-positive (CPS ≥ 1) tumors, leading to the first regulatory approval of pembrolizumab in advanced GC.	First clinical validation of PD-L1 as a predictive biomarker for anti-PD-1 therapy.
KEYNOTE-062 (Shitara et al., 2020) [68]	Phase III randomized trial	763 untreated advanced gastric/GEJ adenocarcinomas with PD-L1 CPS ≥ 1	Showed that clinical benefit from pembrolizumab depends on PD-L1 expression level, with greater benefit observed in tumors with higher CPS values, firmly establishing the clinical importance of CPS scoring.	Validated PD-L1 Combined Positive Score (CPS) as the principal biomarker for patient stratification.
CheckMate 649 (Janjigian et al., 2021) [66]	Phase III randomized trial	1581 patients with untreated HER2-negative advanced gastric/GEJ/oesophageal adenocarcinoma	Nivolumab plus chemotherapy significantly improved overall and progression-free survival, particularly in patients with PD-L1 CPS ≥ 5, establishing chemo-immunotherapy as the first-line standard of care.	Confirmed PD-L1 CPS as the key predictive biomarker guiding first-line immunotherapy.
KEYNOTE-859 (Rha et al., 2023) [67]	Global phase III randomized trial	1579 patients with HER2-negative advanced gastric/GEJ adenocarcinoma	Pembrolizumab plus chemotherapy significantly improved overall survival, progression-free survival and response rate, with increasing benefit according to higher PD-L1 CPS expression.	Contemporary confirmation of PD-L1 CPS as a robust predictive biomarker in first-line GC immunotherapy.
**(** **B) Landmark Studies Underpinning the Selection of dMMR/MSI as a Core Biomarker of Immune Responsiveness in GC**				
**Reference**	**Study Design**	**Cohort**	**Principal Finding**	**Biological/Clinical Milestone**
The Cancer Genome Atlas (TCGA) Research Network, 2014 [56]	Integrated genomic and molecular analysis	295 primary gastric adenocarcinomas	Identified MSI as one of the four molecular subtypes of GC, characterized by a hypermutated phenotype, abundant immune-cell infiltration, and increased immunogenicity.	Established the biological foundation of MSI as a distinct molecular subtype and provided the rationale for immune checkpoint inhibition in GC.
Smyth et al., 2017 (MAGIC exploratory analysis) [69]	Translational analysis of the phase III MAGIC trial	303 resectable gastric and gastro-oesophageal adenocarcinomas	Demonstrated that patients with MSI-H/dMMR tumors had a favorable prognosis and derived little or no benefit from perioperative chemotherapy.	First evidence establishing MSI as both a prognostic biomarker and a potential predictor of chemotherapy response in resectable GC.
Pietrantonio et al., 2019 [70]	Individual patient data meta-analysis (MAGIC, CLASSIC, ARTIST and ITACA-S trials)	1556 patients with resectable GC	Confirmed the favorable prognosis of MSI-H GC and demonstrated the limited benefit of perioperative or adjuvant chemotherapy in this molecular subgroup.	Validation of MSI as a robust prognostic biomarker and a clinically relevant predictor of chemotherapy benefit.
Pietrantonio et al., 2021 [71]	Meta-analysis of randomized immunotherapy trials (including KEYNOTE-061, KEYNOTE-062, CheckMate 649 and JAVELIN Gastric 100)	Patients with advanced gastric/gastro-oesophageal adenocarcinoma enrolled in randomized phase III trials	Demonstrated that MSI-H/dMMR tumors derive the greatest survival benefit from PD-1 blockade compared with microsatellite-stable tumors, irrespective of the specific treatment regimen.	Established MSI as one of the strongest predictive biomarkers for immune checkpoint inhibition in advanced GC.
KEYNOTE-859 (Rha et al., 2023) [67]	Global phase III randomized clinical trial	1579 patients with HER2-negative advanced gastric/gastro-oesophageal junction adenocarcinoma	Confirmed the efficacy of pembrolizumab plus chemotherapy in first-line treatment and reinforced the exceptional responsiveness of the MSI-H subgroup in exploratory biomarker analyzes.	Contemporary clinical validation supporting the role of MSI in selecting patients most likely to benefit from immunotherapy.

**Table 7:** (**A**) Landmark studies underpinning the selection of HER2 as a core actionable biomarker in GC. (**B**) Landmark studies underpinning the development of FGFR2b as an actionable biomarker in GC.

**(A) Landmark Studies Underpinning the Selection of HER2 as a Core Actionable Biomarker in GC**				
**Reference**	**Study Design**	**Cohort**	**Principal Finding**	**Biological/Clinical Milestone**
Tanner et al., 2005 [74]	Clinicopathological study	131 gastric carcinomas	Demonstrated HER2 amplification and overexpression in a biologically distinct subset of GCs and associated HER2 positivity with adverse clinicopathological features.	First large clinicopathological evidence establishing HER2 as a clinically relevant biomarker in GC.
Hofmann et al., 2008 [76]	International pathology validation study	Gastric and gastro-oesophageal junction adenocarcinomas	Developed the GC-specific HER2 immunohistochemical scoring system and validated HER2 assessment using IHC and ISH.	Standardized HER2 testing and established the pathological criteria adopted in clinical practice.
Bang et al. (ToGA), 2010 [75]	International Phase III randomized trial	594 patients with HER2-positive advanced gastric/GEJ adenocarcinoma	Demonstrated that adding trastuzumab to chemotherapy significantly improved overall survival compared with chemotherapy alone.	Established HER2 as the first predictive biomarker and introduced precision medicine into GC.
The Cancer Genome Atlas (TCGA), 2014 [56]	Comprehensive molecular characterization	295 primary gastric adenocarcinomas	Identified HER2 (ERBB2) amplification as a defining feature of the chromosomal instability (CIN) molecular subtype.	Defined the molecular biological context of HER2-positive GC.
LOGiC (Hecht et al., 2016) [77]	International Phase III randomized trial	545 patients with HER2-positive advanced gastric/GEJ adenocarcinoma	Lapatinib combined with chemotherapy failed to significantly improve overall survival despite activity in selected subgroups.	Demonstrated that not all HER2-targeted strategies translate into clinical benefit.
TyTAN (Satoh et al., 2014) [78]	Phase III randomized trial	261 Asian patients with HER2-amplified advanced GC	Lapatinib plus paclitaxel did not significantly improve overall survival in the overall population, although benefit was observed in patients with strong HER2 overexpression (IHC3+).	Highlighted HER2 heterogeneity and the importance of accurate patient selection.
GATSBY (Thuss-Patience et al., 2017) [80]	Adaptive Phase II/III randomized trial	415 previously treated HER2-positive advanced gastric/GEJ adenocarcinomas	Trastuzumab emtansine (T-DM1) failed to improve survival compared with taxane chemotherapy.	Demonstrated the limitations of first-generation HER2 antibody–drug conjugates in GC.
JACOB (Tabernero et al., 2018) [79]	International Phase III randomized trial	780 HER2-positive metastatic gastric/GEJ adenocarcinomas	Addition of pertuzumab to trastuzumab and chemotherapy numerically improved survival but did not meet the primary endpoint.	Refined the concept of dual HER2 blockade in GC.
DESTINY-Gastric01 (Shitara et al., 2020) [68]	Phase II randomized trial	187 patients with previously treated HER2-positive gastric/GEJ adenocarcinoma	Trastuzumab deruxtecan significantly improved objective response rate and overall survival after trastuzumab failure.	Established next-generation HER2 antibody–drug conjugates as an effective therapeutic strategy.
DESTINY-Gastric04 (phase III randomized trial) [81]	Global Phase III randomized trial	494 patients with HER2-positive unresectable/metastatic gastric or GEJ adenocarcinoma after progression on trastuzumab-containing first-line therapy	Trastuzumab deruxtecan significantly improved overall survival compared with ramucirumab plus paclitaxel (median OS, 14.7 vs. 11.4 months; HR 0.70, 95% CI 0.55–0.90; *p* = 0.004), with improved progression-free survival (HR 0.74) and objective response rate (44.3% vs. 29.1%).	Provided randomized Phase III evidence supporting trastuzumab deruxtecan as an effective second-line treatment for HER2-positive advanced gastric/GEJ adenocarcinoma after trastuzumab-based first-line therapy.
**(B) Landmark Studies Underpinning the Development of FGFR2b as an Actionable Biomarker in GC**				
**Reference**	**Study Design**	**Cohort**	**Principal Finding**	**Biological/Clinical Milestone**
Kunii et al., 2008 [82]	Experimental and translational study	GC cell lines and xenograft models	Demonstrated that FGFR2 amplification functions as an oncogenic driver and that inhibition of FGFR2 suppresses tumor growth.	First study identifying FGFR2 amplification as a therapeutically relevant molecular alteration in GC.
Matsumoto et al., 2012 [83]	Clinicopathological study	Primary gastric adenocarcinomas	Showed that FGFR2 amplification and protein overexpression are associated with aggressive clinicopathological features and poor prognosis.	Established the biological and prognostic significance of FGFR2 alterations in GC.
Pearson et al., 2016 [84]	Comprehensive genomic analysis	GC cohorts	Demonstrated marked intratumoral heterogeneity of FGFR2 amplification and highlighted its implications for biomarker assessment and therapeutic resistance.	Defined the complexity of FGFR2 as a predictive biomarker and emphasized sampling considerations.
FPA144-001 (Catenacci et al., 2020) [85]	First-in-human Phase I study	Advanced FGFR2b-positive gastric/GEJ adenocarcinoma	Demonstrated the feasibility, safety and preliminary antitumor activity of the FGFR2b-specific monoclonal antibody bemarituzumab.	First clinical proof-of-concept for selective FGFR2b inhibition.
FIGHT (Wainberg et al., 2022) [86]	Randomized double-blind Phase II trial	155 patients with HER2-negative FGFR2b-positive advanced gastric/GEJ adenocarcinoma	Bemarituzumab plus mFOLFOX significantly improved progression-free survival, overall survival and objective response rate compared with chemotherapy alone, with greatest benefit in tumors showing high FGFR2b expression.	Established FGFR2b as the second clinically actionable biomarker in GC after HER2.
FIGHT final analysis (Wainberg et al., 2024) [87]	Final efficacy analysis of Phase II trial	FGFR2b-positive advanced gastric/GEJ adenocarcinoma	Confirmed durable survival benefit, particularly in patients with ≥10% FGFR2b-positive tumor cells, supporting biomarker enrichment strategies.	Validated FGFR2b expression level as a clinically meaningful predictive biomarker.
FORTITUDE-101 [88]	Global randomized Phase III trial	First-line FGFR2b-overexpressing advanced gastric/GEJ adenocarcinoma	Ongoing confirmatory trial evaluating bemarituzumab plus chemotherapy and nivolumab versus standard therapy.	Expected to establish FGFR2b-directed therapy as a first-line standard of care.

**Table 8:** Clinically relevant gastric cancer biomarkers: testing and clinical utility. Summary of the principal biomarkers, their testing methods, interpretation criteria, clinical roles, therapeutic implications, and levels of evidence.

Biomarker	Biological Domain	Method/Assay	Cut-Off/Interpretation	Clinical Role	Therapy/Level of Evidence
HER2	Actionable pathways	IHC ± ISH; HERCEPTEST	IHC 3+ or IHC 2+/ISH+	Predictive	Trastuzumab; T-DXd; Clinical implementation
PD-L1	Immune evasion	IHC; CPS; 22C3/28-8	CPS (treatment-specific thresholds)	Predictive	PD-1 inhibition; Clinical implementation
MSI/dMMR	Immune evasion	MMR IHC ± PCR/NGS	MSI-H or MMR protein loss	Predictive/molecular classification	Immune-checkpoint inhibitors; Clinical implementation
CLDN18.2	Lineage/actionable	IHC; VENTANA 43-14A	≥75% tumor cells with 2+/3+ membranous staining	Predictive	Zolbetuximab; Clinical implementation
FGFR2b	Actionable pathways	IHC; clinical-trial assays	≥10% tumor cells with 2+/3+ staining	Emerging predictive	Bemarituzumab; Clinical evidence
NTRK fusions	Genomic alterations	DNA/RNA NGS	NTRK1/2/3 fusion present	Predictive	TRK inhibitors; Clinical implementation
BRAF V600E	Genomic alterations	NGS/mutation assay	BRAF V600E present	Predictive	BRAF/MEK inhibition; Clinical implementation
RET fusions	Genomic alterations	DNA/RNA NGS	RET fusion present	Predictive	RET inhibitors; Clinical implementation
TMB-H	Genomic/immune	NGS	≥10 mutations/Mb	Predictive	Pembrolizumab in eligible tumors; Clinical implementation
VSIG1	Lineage identity	IHC ± molecular	No validated cutoff	Biological/research	No established therapy; Research
LGR5	Stemness	IHC/molecular	No validated cutoff	Biological/prognostic research	No established therapy; Research
CD44v9	Stemness/plasticity	IHC	No validated cutoff	Biological/prognostic research	No established therapy; Research
NNMT	Metabolic/epigenetic	IHC/molecular	No validated cutoff	Emerging research	No established therapy; Preclinical

## Data Availability

No new data were created or analyzed in this study. Data sharing is not applicable to this article.

## Supplementary Materials

The following supporting information can be downloaded at: https://www.mdpi.com/2072-6694/18/17/2757/s1, Table S1. Complete PubMed/MEDLINE Search Strategy for Gastric Cancer Biomarker Studies. Table S2. Study quality and risk-of-bias assessment framework.
