# Supplementary material for: Gastric Cancer Biomarkers: From Cellular Identity to Precision Oncology
Source: Cancers (Basel). 2026 Aug 25;18(17):2757. doi: 10.3390/cancers18172757 (PMC13572867; doi:10.3390/cancers18172757)
Supplement: Supplementary file 1 [file cancers-18-02757-s001.zip › cancers-4522452-supplementary.pdf]

Supplementary Table S1. Complete PubMed/MEDLINE Search Strategy for Gastric Cancer Biomarker Studies

Search block	PubMed/MEDLINE terms
Gastric cancer	*("Stomach Neoplasms"[Mesh] OR "gastric cancer"[Title/Abstract] OR "gastric carcinoma"[Title/Abstract] OR "gastric adenocarcinoma"[Title/Abstract] OR "stomach cancer"[Title/Abstract] OR "gastric neoplasm"[Title/Abstract])**
Biomarkers / clinical utility	*("Biomarkers"[Mesh] OR biomarker[Title/Abstract] OR "molecular marker*"[Title/Abstract] OR "biological marker*"[Title/Abstract] OR "predictive marker*"[Title/Abstract] OR "prognostic marker*"[Title/Abstract] OR "diagnostic marker*"[Title/Abstract] OR "treatment selection"[Title/Abstract])**
Gastric lineage / differentiation	(CLDN18[Title/Abstract] OR CLDN18.2[Title/Abstract] OR VSIG1[Title/Abstract] OR MUC5AC[Title/Abstract] OR MUC6[Title/Abstract] OR TFF1[Title/Abstract] OR "trefoil factor 1"[Title/Abstract] OR "pepsinogen C"[Title/Abstract] OR PGC[Title/Abstract])
Stemness / plasticity	(LGR5[Title/Abstract] OR CD44[Title/Abstract] OR CD44v9[Title/Abstract] OR "CD44 variant"[Title/Abstract] OR ALDH1[Title/Abstract] OR ALDH1A1[Title/Abstract] OR SOX2[Title/Abstract] OR OCT4[Title/Abstract] OR POU5F1[Title/Abstract] OR NANOG[Title/Abstract] OR BMI1[Title/Abstract] OR EpCAM[Title/Abstract] OR EPCAM[Title/Abstract])
EMT / epithelial plasticity	("E-cadherin"[Title/Abstract] OR CDH1[Title/Abstract] OR SNAIL[Title/Abstract] OR SNAI1[Title/Abstract] OR ZEB1[Title/Abstract] OR TWIST1[Title/Abstract] OR "N-cadherin"[Title/Abstract] OR CDH2[Title/Abstract] OR vimentin[Title/Abstract] OR VIM[Title/Abstract] OR "beta-catenin"[Title/Abstract] OR CTNNB1[Title/Abstract] OR "miR-200"[Title/Abstract])
Immune evasion / immune response	("PD-L1"[Title/Abstract] OR "programmed death ligand 1"[Title/Abstract] OR CD274[Title/Abstract] OR "PD-L2"[Title/Abstract] OR PDCD1LG2[Title/Abstract] OR "PD-1"[Title/Abstract] OR PDCD1[Title/Abstract] OR "mismatch repair deficiency"[Title/Abstract] OR dMMR[Title/Abstract] OR "microsatellite instability"[Title/Abstract] OR MSI[Title/Abstract] OR "MSI-H"[Title/Abstract] OR "tumor mutational burden"[Title/Abstract] OR TMB[Title/Abstract] OR EBV[Title/Abstract] OR "Epstein-Barr virus"[Title/Abstract] OR "CTLA-4"[Title/Abstract] OR CTLA4[Title/Abstract] OR "LAG-3"[Title/Abstract] OR LAG3[Title/Abstract] OR TIGIT[Title/Abstract])
Actionable oncogenic pathways	(HER2[Title/Abstract] OR ERBB2[Title/Abstract] OR FGFR2[Title/Abstract] OR FGFR2b[Title/Abstract] OR MET[Title/Abstract] OR EGFR[Title/Abstract] OR PIK3CA[Title/Abstract] OR KRAS[Title/Abstract] OR BRAF[Title/Abstract] OR "BRAF V600E"[Title/Abstract] OR NTRK[Title/Abstract] OR NTRK1[Title/Abstract] OR NTRK2[Title/Abstract] OR NTRK3[Title/Abstract] OR RET[Title/Abstract])
Metabolic / epigenetic biomarker	(NNMT[Title/Abstract] OR "nicotinamide N-methyltransferase"[Title/Abstract])

Supplementary Table S2. Study quality and risk-of-bias assessment framework.

Study design	Main quality domains assessed	Key sources of potential bias
Randomized clinical trials	Randomization, allocation concealment, blinding, completeness of outcome data, selective reporting	Selection, performance, attrition, reporting bias
Prospective observational studies	Cohort selection, exposure/biomarker assessment, outcome definition, follow-up completeness, confounding control	Selection, measurement, confounding, attrition bias
Retrospective observational studies	Patient selection, data completeness, biomarker assessment, outcome ascertainment, confounding control	Selection, information, confounding bias
Diagnostic/biomarker studies	Patient spectrum, reference standard, index-test methodology, blinding, applicability	Selection, measurement, verification, spectrum bias
Systematic reviews/meta-analyses	Search completeness, study selection, methodological quality of included studies, heterogeneity, publication bias	Selection, reporting, publication bias, heterogeneity
Preclinical studies	Experimental design, model selection, controls, replication, outcome measurement	Model-related, experimental, reporting bias
